# A Hitchhiker's guide to RNA–RNA structure and interaction prediction tools

**DOI:** 10.1093/bib/bbad421

**Published:** 2023-12-01

**Authors:** Francis Yew Fu Tieng, Muhammad-Redha Abdullah-Zawawi, Nur Alyaa Afifah Md Shahri, Zeti-Azura Mohamed-Hussein, Learn-Han Lee, Nurul-Syakima Ab Mutalib

**Affiliations:** UKM Medical Molecular Biology Institute (UMBI), Universiti Kebangsaan Malaysia (UKM), Kuala Lumpur 56000, Malaysia; UKM Medical Molecular Biology Institute (UMBI), Universiti Kebangsaan Malaysia (UKM), Kuala Lumpur 56000, Malaysia; UKM Medical Molecular Biology Institute (UMBI), Universiti Kebangsaan Malaysia (UKM), Kuala Lumpur 56000, Malaysia; Institute of Systems Biology (INBIOSIS), UKM, Selangor 43600, Malaysia; Department of Applied Physics, Faculty of Science and Technology, UKM, Selangor 43600, Malaysia; Sunway Microbiomics Centre, School of Medical and Life Sciences, Sunway University, Sunway City 47500, Malaysia; Novel Bacteria and Drug Discovery Research Group, Microbiome and Bioresource Research Strength, Jeffrey Cheah School of Medicine and Health Sciences, Monash University of Malaysia, Selangor 47500, Malaysia; UKM Medical Molecular Biology Institute (UMBI), Universiti Kebangsaan Malaysia (UKM), Kuala Lumpur 56000, Malaysia; Novel Bacteria and Drug Discovery Research Group, Microbiome and Bioresource Research Strength, Jeffrey Cheah School of Medicine and Health Sciences, Monash University of Malaysia, Selangor 47500, Malaysia; Faculty of Health Sciences, UKM, Kuala Lumpur 50300, Malaysia

**Keywords:** RNA–RNA interaction prediction, RNA interactome, RNA structure prediction, computational tools

## Abstract

RNA biology has risen to prominence after a remarkable discovery of diverse functions of noncoding RNA (ncRNA). Most untranslated transcripts often exert their regulatory functions into RNA–RNA complexes via base pairing with complementary sequences in other RNAs. An interplay between RNAs is essential, as it possesses various functional roles in human cells, including genetic translation, RNA splicing, editing, ribosomal RNA maturation, RNA degradation and the regulation of metabolic pathways/riboswitches. Moreover, the pervasive transcription of the human genome allows for the discovery of novel genomic functions via RNA interactome investigation. The advancement of experimental procedures has resulted in an explosion of documented data, necessitating the development of efficient and precise computational tools and algorithms. This review provides an extensive update on RNA–RNA interaction (RRI) analysis via thermodynamic- and comparative-based RNA secondary structure prediction (RSP) and RNA–RNA interaction prediction (RIP) tools and their general functions. We also highlighted the current knowledge of RRIs and the limitations of RNA interactome mapping via experimental data. Then, the gap between RSP and RIP, the importance of RNA homologues, the relationship between pseudoknots, and RNA folding thermodynamics are discussed. It is hoped that these emerging prediction tools will deepen the understanding of RNA-associated interactions in human diseases and hasten treatment processes.

## INTRODUCTION

More than 60 years ago, the central dogma of molecular biology was first introduced by Francis Crick as a model to describe the transfer of genetic information from DNA to protein [1]. Since then, several attempts have been made to interpret the composition of RNA subtypes in the human genome and their roles in protein synthesis [2, 3]. Typically, Watson–Crick base-pairing is known to maintain the genetic continuity of RNA replication, and encoded proteins are not involved as catalysts [1, 4]. The adaptability of RNA molecules has spawned the ‘RNA World’ hypothesis, in which RNA replication-based evolution takes precedence over DNA-centred evolution and protein synthesis [2, 5–7]. The ‘RNA World’ hypothesis depicts the possibility of storing genetic material via RNA alone and its ability to self-replicate as the primary source of catalytic mechanisms without the involvement of proteins [8–17]. Since the discovery of protein-encoding messenger RNA (mRNA) in the 1960s, it has received a great deal of attention due to its critical function in protein synthesis and is considered the inevitable intermediary necessity in producing proteins [18]. Nevertheless, high-throughput sequencing platforms create a paradigm shift, as over 90% of the human genome is transcribed into RNA [18, 19]. Of all, 2% of the RNA in the genome encodes proteins, while the remaining is easily transcribed into nonprotein-encoded RNA (also known as noncoding RNA or ncRNA) molecules [20–24]. In summary, advances in sequencing technology have enabled the discovery of ncRNAs, bringing RNA biology to the forefront and revealing the intricate role of ncRNAs in human cells [25–28].

Noncoding RNAs (ncRNAs) are RNA molecules that are not translated into proteins. Their length can be classified into three categories: (i) short (19 to 31 nucleotides), (ii) mid (20 to 200 nucleotides) and (iii) long (>200 nucleotides) [29]. Among them, microRNAs (miRNAs) are the most well-studied short ncRNAs, acting as supplementary posttranscriptional regulators and ‘buffers’ that maintain the robustness of biological systems [30, 31]. In contrast, long ncRNAs (lncRNAs) are the largest ncRNA subtype, with approximately 55 000 genes along the genome [32, 33].

Despite extensive functional studies, the molecular mechanisms of ncRNA-centric roles remain elusive and require advances in experimental biomedicine [34–36]. However, emerging RNA–RNA interaction (RRI) tools offer promise in reducing experimental efforts. Understanding these mechanisms requires investigating ncRNA interactions with cellular components such as proteins, DNA sites and other RNAs [37]. Remarkably, numerous classical ncRNAs communicate with other RNA subtypes, either directly via base pairing or indirectly via protein intermediates. Examples include transfer RNA-messenger RNA (tRNA–mRNA) interactions to translate genetic code; miRNA–mRNA interactions to stimulate mRNA degradation; and mRNA–protein interactions involving RNA splicing, editing and ribosomal RNA maturation [38–41]. These findings imply that RRIs portray a universal strategy utilized by many ncRNAs, and completely mapping these interactions could provide insight into ncRNA functions and mechanisms. The RNA interactome has emerged as a central component of many regulatory processes, prompting extensive research from both wet lab and computational researchers [42–45]. Nonetheless, mapping RRIs remains challenging, as current methods struggle to identify and differentiate between direct and indirect RRIs and may have limited resolution for specific RNA examination.

## TYPES OF INTERACTIONS

RNA molecules are not just passive carriers of genetic information; they actively participate in various cellular processes through their interactions with other molecules [46]. Understanding these roles and interactions is crucial for advancing our knowledge of cellular biology. RNA molecules interact with other RNAs, proteins and DNA to carry out their functions.

RNA–DNA interactions are essential for several biological processes. One of the most well-known examples is transcription, where an RNA molecule is synthesised based on the DNA template. Another example is the process of reverse transcription in retroviruses, where viral RNA is reverse transcribed into DNA. For instance, in RNA interference (RNAi), small RNA molecules can bind to complementary sequences in mRNA molecules, leading to their degradation and thus preventing their translation into proteins [47]. Another example is the clustered regularly interspaced short palindromic repeats system, a bacterial defense mechanism that has been adapted for genome editing. In this Nobel-prize winner system, RNA molecules guide the Cas9 nuclease to specific locations in the DNA, allowing precise cuts to be made [48]. More recent studies have also highlighted the role of ncRNAs in regulating chromatin architecture via interaction with DNA or chromatin-associated proteins to modulate gene expression. Some ncRNAs function through the formation of R-loops with the complementary sequence from their transcribed loci and affect local gene expression [49].

RNA–protein interactions are fundamental to cellular processes and play a crucial role in the life cycle of an RNA molecule, from its synthesis and processing to its eventual function in protein synthesis. Proteins can bind to RNA to form ribonucleoprotein complexes, which are involved in various aspects of RNA metabolism, including splicing, polyadenylation, stability, transport, and translation [50]. The spliceosome, a large ribonucleoprotein complex, is responsible for removing introns from pre-mRNA, a process known as splicing, and is crucial for the maturation of mRNA molecules and their subsequent translation into proteins [51]. RNA–protein interactions also play a role in polyadenylation, the addition of a poly(A) tail to the 3′ end of an mRNA molecule that enhances the stability of the mRNA and facilitates its export from the nucleus and transport within the cell [52]. During protein translation, mRNA molecules interact with ribosomes, which are themselves ribonucleoprotein complexes, to synthesize proteins based on the sequence of the mRNA that determines the sequence of amino acids in the protein [53].

RNA also interacts with other RNA. For instance, RNA molecules can form complex secondary and tertiary structures through interactions with other RNA molecules, whereby these structures are critical for the function of many types of RNA, including ribosomal RNA (rRNA), transfer RNA (tRNA) and mRNA [54]. In the ribosome, which is a complex of rRNA and proteins, mRNA and tRNA interact to facilitate protein synthesis. The rRNA provides the structural framework for the ribosome and contributes to its catalytic activity [55]. RRIs also play a role in the regulation of gene expression. For instance, miRNAs can base-pair with target mRNAs to repress their translation or induce their degradation [56]. Dysregulation of RRIs can lead to various diseases. For example, mutations that affect the secondary structure of RNA can disrupt normal RRIs and lead to diseases such as cancer [57]. Understanding these interactions is crucial, as they play a significant role in cellular processes, and their dysregulation can lead to various diseases. Therefore, tools that can predict and analyse these interactions are of great importance in advancing our knowledge of cellular biology and developing therapeutic strategies.

### Types of RNA–RNA interactions

**Figure 1:**
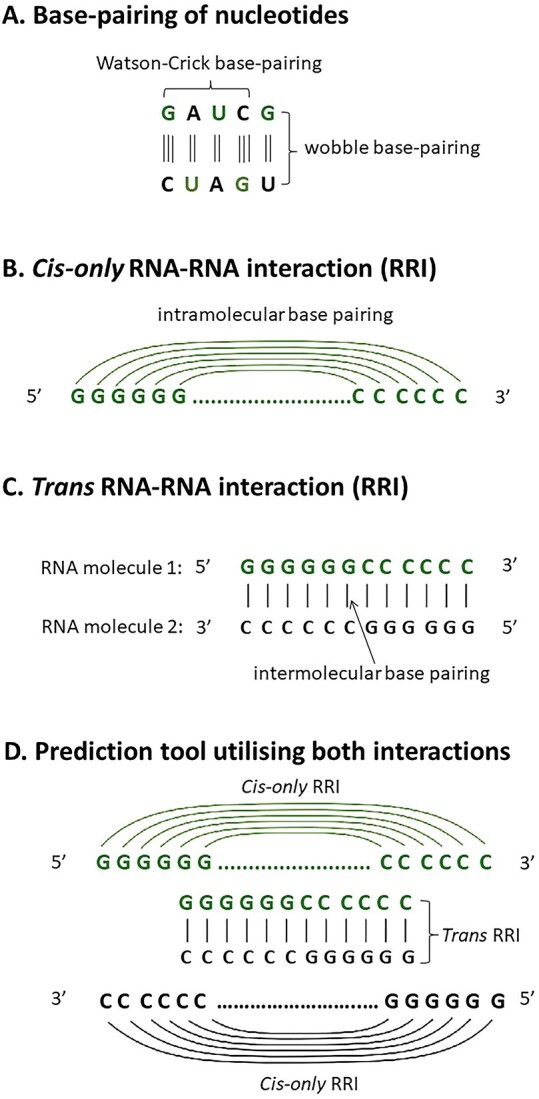
Potential interactions in RNA molecules. (**A**) Possible base-pairing of nucleotides. (**B**) cis-only RRI (intramolecular base-pairing) within a single RNA molecule. (**C**) Trans RRI (intermolecular base-pairing) between two identical RNA molecules. (**D**) Situation in concatenation-based prediction tool, where both RRI types are involved. Inter- and intramolecular base pairs are indicated by vertical pipe symbols and arches, respectively (adapted from [25]).

There are two main types of interactions in RNA molecules, namely, *cis-only* and *trans* RRIs (Figure 1). The former is defined as the intramolecular base pairing between nucleotides within a single RNA molecule (Figure 1**B**) [58]. It permits canonical Watson–Crick base-pairing between {adenine (A) and uracil (U)} and {guanine (G) and cytosine (C)} and non-Watson–Crick/wobble base-pairing between {guanine (G) and uracil (U)} (formed by edge-to-edge hydrogen bonding interactions between the bases) (Figure 1**A**) [59–61]. The intramolecular RRI aids in the formation of short double-stranded helices and allows folding into specific 3D structures such as tRNA and mRNA, which form the basis for molecular recognition events [62, 63].

On the other hand, *trans* RRI is made up of two or more RNAs that interact intermolecularly via Watson–Crick base pairing, wobble base pairing or helical stacking (Figure 1**C**) [64, 65]. miRNAs, for example, can target the 3′ untranslated regions (3’ UTRs) of mRNAs [66–68], whereas spliceosomal small nuclear RNAs (snRNAs) recognize the intronic regions of pre-mRNAs [69, 70]. Duplex formation through base pairing of complementary nucleotides leads to naturally occurring RRIs. They are crucial for various processes, including RNA cleavage, RNA editing, RNA modification, RNA splicing, RNA translation, suppression of RNA translation and RNA degradation [71–75]. Additionally, base-pair interactions are crucial for semiconservative replication, energetically favourable arrangement of base pairs, and the formation of helical RNA structures [76]. Intramolecular interactions lead to the formation of RNA secondary structures, which is why researchers commonly refer to the prediction of *cis-only* RRIs as the method for RNA structure prediction (RSP). To summarise, intramolecular interactions form secondary RNA structures (*cis-only* RRI), while intermolecular interactions occur when two individual RNAs interact (*trans* RRI).

Predicting RRIs based solely on intra- or intermolecular interactions presents significant challenges due to the diverse conformations [77, 78] and conformational changes of RNA molecules [79–81]. Complexities also arise from the three-dimensional folding, secondary structures [82] and tertiary interactions of RNA molecules [83]. Therefore, focusing exclusively on one type of RNA interaction may result in the oversight of crucial interactions occurring across different regions of an RNA molecule [84]. Nonetheless, concatenating both intra- and intermolecular RNA interactions (Figure 1**D**) permits a more comprehensive analysis, capturing a broader range of interactions and revealing complex RNA networks. This integrated approach provides a more realistic representation of RRIs in biological systems and offers insights into their contribution to overall RNA architecture. Utilizing both types of interactions for prediction provides a more robust and holistic framework compared to relying on either one alone.

RRIs are modelled at various levels of complexity, depending on their common and distinguishing features, which are translated into sophisticated computational algorithms. Complexity refers to the intricacy and sophistication of the computational approach used to model RRIs. However, current RRI models cannot account for real-time biological and chemical information in the cellular environment, except at a coarser level of detail [85]. These models typically focus on sequence complementarity, thermodynamic stability, or structural motifs, which may not fully capture the intricacies of the cellular context [86]. Using RSP-like algorithm tools could facilitate RRI prediction (RIP) by providing reliable information on interacting nucleotide positions, revealing potential biological roles and regulatory mechanisms of mRNAs and ncRNAs [87]. In short, there is a need for RSP-like algorithms to better understand RNA sequences and their interactions in real time, improving RIP models and gaining deeper insights into their biological significance.

## RNA–RNA INTERACTION MAPPING VIA EXPERIMENTAL DATA: LIMITATIONS AND TECHNIQUES

The secondary structure of ncRNA serves as a scaffold for the tertiary structure and facilitates catalytic and ligand binding interactions with various RNAs [33, 44, 88]. RIP tools use similar ideas and algorithms to predict RNA secondary structures. X-ray crystallography (single crystal X-ray diffraction (XRD)) and nuclear magnetic resonance (NMR) spectroscopy are the most accurate and robust conventional methods for detecting three-dimensional (3D) RNA structures [89, 90]. Although XRD provides high atomic resolution with no size limitations, crystallizing 3D RNA structures is challenging. Conversely, NMR excels when crystallization is impossible and provides solution state dynamics but has limitations on molecular weights (<50 kDa) [91]. Combining XRD and NMR results in a more accurate structure determination method, providing ncRNA structural information at a single base-pair resolution [92, 93]. Nonetheless, their widespread application is hampered by high experimental costs, low throughput, limited ncRNA resolution measurements and structure detection *in vitro*, difficulty in translating to *in vivo* conformation, and < 0.001% of ncRNAs identified from experimental data [94].

Numerous sequencing-based systems have been developed over the last decade for the experimental identification of RNA interactomes. However, current RRI mapping methods, such as RNA interactome analysis and sequencing (RIA-Seq) and RNA antisense purification (RAP)-Seq, do not directly assay RNA interactomes [95, 96]. Instead, they rely on anchored RNAs and molecular perturbations to identify interaction targets of specific RNAs [97]. This one-RNA-at-a-time approach makes it challenging to comprehensively identify all RRIs. Following this, several high-throughput techniques, including PARIS [98], SPLASH [99], LIGR-Seq [100] and MARIO [97], have been introduced. They map the entire RNA interactomes *in vivo* besides identifying interacting partners of specific target RNAs at a larger scale. Online databases such as RAID v2.0 [101], NPinter [102–104], RNAinter [105, 106] and RISE [107] organise and classify these RRIs based on curated data from various sources (bibliometrics, experimental data, etc.). Nevertheless, a complete picture of human RNA-associated interactions is lacking, with most observed interactions associated with ribosomal and small RNAs rather than ncRNAs. Tissue-specific expression patterns of RNAs require numerous repetitions of *in vivo* experiments to detect genome-wide RNA interactomes [20, 108]. Therefore, computational RIP methods remain indispensable compared to experimental approaches.

## STATE-OF-THE-ART APPROACHES FOR RNA STRUCTURE AND INTERACTOME PREDICTION

Computational prediction methods are widely used for identifying RRIs. The discovery of the minimum free energy (MFE) structure of RNA sequences has garnered attention due to its association with RNA secondary structures and folding stability. The MFE of an RNA includes the sequence length, nucleotide content/composition and nucleotide order/arrangement [109]. Longer RNA sequences tend to be more stable due to stacking and hydrogen bond interactions [110]. The composition of nucleotides also influences RNA stability; G-C-rich sequences are more durable than A-U-rich sequences due to additional hydrogen bonds. The specific arrangement of nucleotides, including loop numbers and double helix conformations, contributes to folding structure stability [109].

**Figure 2:**
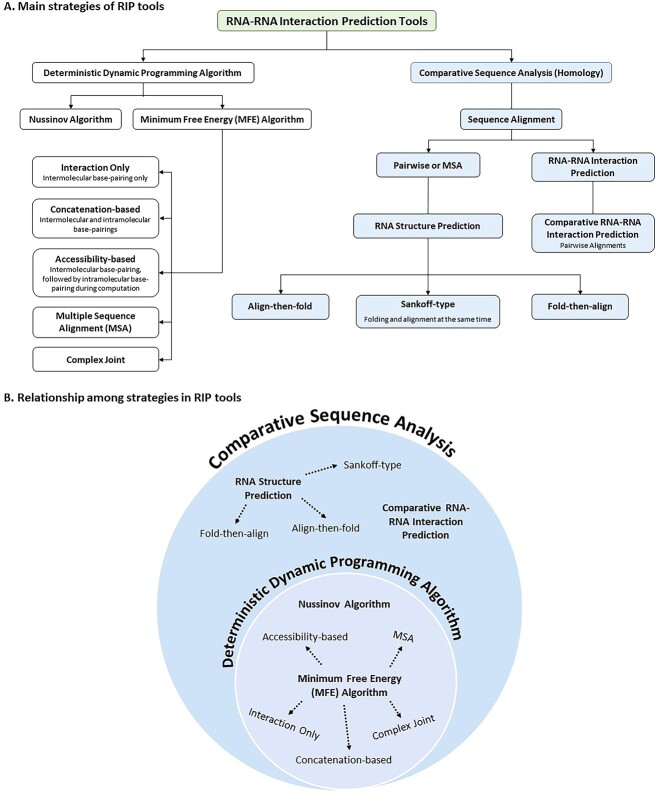
Foundation of RNA–RNA interaction prediction tools. (**A**) Two core strategies, namely, deterministic dynamic programming algorithm and comparative sequence analysis. (**B**) Venn diagram portraying the relationships between these strategies and emphasizing the overlap, demonstrating their interconnectedness.

This review aimed to summarise popular computational prediction tools for RIP based on two main strategies: deterministic dynamic programming (DDP) approach and comparative sequence analysis (homology), as illustrated in Figure 2**A** [85–87, 111–113]. This landscape reflects the growing interest and extensive research in the field of RIP. Figure 2**B** showcases the relationships between these two strategies.

### Deterministic dynamic programming algorithm for individual RNA structure and RNA–RNA interaction prediction

The DDP algorithm is a popular and accurate type of RIP that relies on the thermodynamics model. It uses free energy minimization to predict RNA secondary structure based on a single sequence with a known function as an input [114]. DDP involves chemically altering nucleotides at Watson-Crick pairing sites in folded RNA using chemicals such as dimethyl sulfate and kethoxal. It is known as a "score-based method" that interprets the native RNA structure with a minimum/maximum total score of RNA folding prediction.

This approach relies on experimental approximations to account for the influence of sequence on stability for different RNA motifs. However, it does not account for pseudoknots, which are RNA structures formed by two nonnested base pairs. The nearest-neighbour model considers directly neighbouring bases and base pairs for each interaction [115, 116], utilizing loop-specific energy contributions to determine loop type- and context-specific contributions to the RNA structure [114, 117, 118].

#### Nussinov algorithm

The application of DDP in RSP ensures efficient computation [119, 120], producing consistent and identical results for identifying the lowest free energy structure. DDP simplifies complex RNA structures into simpler substructures through mathematical optimization and computer programming [119]. The DDP algorithm can be divided into several examples, as reported in Figure 2**A**. The Nussinov algorithm is the first DDP algorithm that efficiently predicts the optimal folding state of an RNA molecule by computing the maximum number of base-pairings [121]. However, it has several biases that can be noted as limitations. For instance, it (i) disregards differences in base-pairing strengths; the influence of loop sizes, base-pair stackings, loop context, multiloop, and pseudoknot formations on stability; (ii) lacks approximation-based prediction algorithms that cause the inability to predict pseudoknotted helices; (iii) does not consider folding kinetics, which does not apply to secondary RNA structures; (iv) exhibits asymmetry in the distribution of unpaired nucleotides, leading to destabilization of multibranch loops/helical junctions; (v) shows discontinuity in the formed base pairs; and (vi) is unable to create stem regions, thereby reducing its prediction accuracy [114, 122].

To address this, a minimum free energy (MFE) algorithm based on the Nussinov algorithm and nearest-neighbour model was proposed by Zuker in 1981 [123].

#### Minimum free energy algorithm

**Figure 3:**
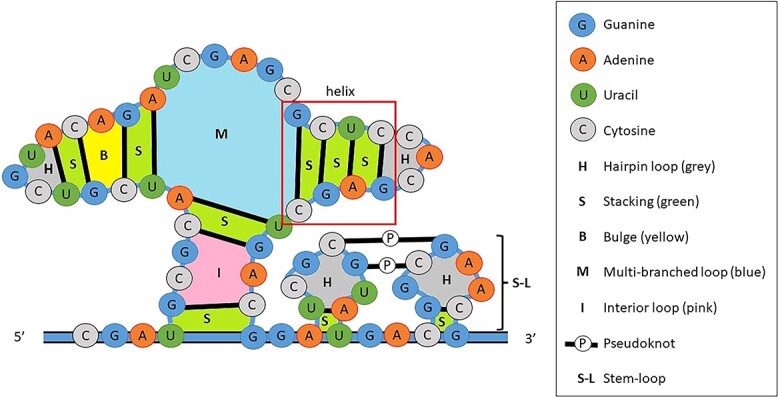
Loop decomposition of a nested RNA structure into hairpin loops (no enclosed base pairs), stackings (adjacent enclosed base pairs), bulges (only one side adjacent to enclosed base pair), multibranched loops (more than one directly enclosed base pair), interior loops (no stacked enclosed base pairs), pseudoknots (nucleotides in a loop pair with a region outside the helices that close the loop) and stem-loops (combination of the stem, double helix, and a loop) (adapted from [256]).

MFE algorithms, based on DPP, compute a series of complex free-energy parameters obtained from experimental methods. One example is the optical melting experiment that measures the thermodynamics of nucleotides. These algorithms breakdown a secondary RNA structure into substructures known as nearest-neighbour loops (Figure 3). The free energy of each nearest-neighbour loop is computed by adding its specific free energy parameters. The MFE approach can be categorised into four subclasses based on criteria, including intramolecular base pairs (internal structure), neglect of intramolecular structure, accessibility of the binding region, and the ability to predict the joint secondary structure of RNA duplexes [124].

This review provides an overview of MFE algorithms derived from RSP and used in RIP tools to predict the RNA interactome in Tables 1–3 [42, 113, 114, 125]. It outlines the main prediction and output strategies employed by each algorithm. ‘Conservation’ indicates whether the prediction tools accept sequence alignments as input, which can help in identifying conserved regions within RNA molecules. ‘Suboptimal’ indicates whether the algorithms report suboptimal results in addition to a single MFE prediction. This feature allows the exploration of alternative RNA secondary structures with lower free energy but remain biologically relevant. The length of the interaction estimates the size of the predicted RNA–RNA helices, categorized as short (≤12 base pairs) or long (>12 base pairs). Additionally, the table distinguishes between local interactions and global predictions. ‘Local interactions’ involve single interactions with gaps and bulges, limited to a few base pairs. These predictions focus on aligning local regions with high similarity. In contrast, ‘global predictions’ span the entire RNA sequence, including multiple instances of local interactions separated by longer regions lacking intermolecular base pairs.

**Table 1:** Interaction-only RIP tools based on MFE algorithms

Characteristic			Interaction-only RIP Tool	Description	Input	Output	Applicable Species	Active (T)/Inactive (F)
Conservation	Suboptimal Prediction	Local Interaction Length	RNAduplex (RNA–RNA) [125]	A tool for pairwise alignment that predicts conserved RRIs between two alignments, typically using RNAcofold for general cases	Two alignments of RNA sequences in CLUSTAL format with an equal number of sequences and the same order	Prediction of the conserved RRIs between 2 alignments Prediction of inter−molecular base pairs only Prediction of binding sites Computation of the optimal and suboptimal secondary structures upon hybridization of two RNA sequences	Any species (more accurate for humans and mice)	T
			RNAaliduplex (RNA–RNA) [125]	An MSA version of RNAduplex, used to predict conserved RRIs between two alignments and the stability of RNA duplex by base-pairing	Multiple RNA sequences in CLUSTAL format (equal number of sequences and same order)	Prediction of intermolecular base pair via RNAcofold Prediction of the optimal and suboptimal binding sites, hybridization energies and the corresponding structures Prediction of the evolutionary conserved binding sites via alignments	Any species (more accurate for humans and mice)	T
	No suboptimal		TargetRNA (sncRNA-mRNA) [127]	A web-based tool that predicts mRNA targets of small ncRNAs in bacteria, often used with RNATarget for reverse searching	A genomic sequence that may correspond to an sRNA gene	Prediction of the mRNA targets of base-pairing sRNAs Calculation of hybridization scores for the sRNA sequence with each message in a genome Determination of statistically significant potential sRNA-mRNA interaction (similar to the RNAhybrid algorithm)	Bacteria	T
No Conservation	Suboptimal Prediction		RIsearch (ncRNA-RNA) [129]	A fast RRI search using simplified nearest-neighbour energy across whole sequences, offering similar accuracy with 2.4x speed improvement compared to RNAplex, with a genome-wide screening prefilter to reduce binding sites	RNA sequences in FASTA format	Fast identification of ncRNA-RNA duplexes Identification of near-complementary base-pairing identification Prediction of near-complementary duplexes Detection of potential RNA–RNA duplexes	Human	T
			GUUGle (miRNA) [130]	A utility tool for fast exact matching under RNA complementary rules, including G–U base pairing, serving as an effective filter before computationally intensive tasks like miRNA target prediction	A set of target sequences and a set of query sequences in various formats (nbrf/pir; codata; textual; unambiguous pure nucleotide; unambiguous pure RNA sequence; FASTA; FASTQ; XML; EMBL or ig), with a length threshold (k)	Identification of all matches (under RNA rules) between the target and the reverse of the query sequences that have k or more consecutive base pairs Quick determination of potential regions of inter- or intramolecular hybridization to speed up the prediction of secondary structure or complex formation Adaptation to be used as a precomputed suffix array of the positive/forward sequence set	All species	T
	No Suboptimal	Global interaction length	RNAhybrid (miRNA–mRNA) [126]	A fast and effective pairwise prediction tool for miRNA/target duplexes, often used as a web service for remote calls from user-implemented programs	Two RNA sequences, which will be handled simultaneously	Prediction of previous or new miRNA targets Prediction of the miRNA targets in the coding sequence Large databases search for long potential target sequences and solve addressed problems directly and effectively without having to use makeshift adaptations of existing RSP programs	All species	T

G: guanine; lncRNA: long noncoding RNA; miRNA: microRNA; mRNA: messenger RNA; MSA: multiple sequence alignment; ncRNA: noncoding RNA; RIP: RNA–RNA interaction prediction; RRI: RNA–RNA interaction; RNA: ribonucleic acid; RSP: RNA structure prediction; sRNA: small RNA; U: uracil

**Table 2:** Accessibility-based RIP tools based on MFE algorithms

Characteristic			Accessibility-based RIP Tool	Description	Input	Output	Applicable Species	Active (T)/ Inactive (F)
Conservation	Suboptimal Prediction	Local Interaction Length	LncRRIsearch (lncRNA–lncRNA and lncRNA–mRNA) [258]	A web server for pairwise alignment and comprehensive prediction of human and mouse lncRNA–lncRNA and lncRNA–mRNA interaction, including tissue-specific or subcellular localised lncRNA interactions	Longest mRNA and lncRNA transcript sequences excluding excluded transcripts in the pseudoautosomal region on the Y-chromosome	Prediction of the RRIs (seed-and-extension approach), accessible energy and hybridization energy Expression analysis for tissue-specific RRIs Prediction of the subcellular localised RRIs RIblast program for comprehensive RIP	Human, animal	T
			RIblast (sRNA and lncRNA TINCR) [141]	The fastest software for pairwise alignment and large-scale lncRNA datasets by parallelization method	A query RNA and a target RNA	Prediction of the intermolecular base pair only Prediction of the sRNA target Prediction of the lncRNA TINCR target	All species	T
			TargetRNA2 (sRNA-mRNA) [142]	A tool that identifies targets of srRNAs in bacteria, allows incorporation of RNA-Seq data, and comparative RIP with MSA	Sequence of an sRNA in FASTA format and the name of a sequenced bacterial replicon	Identification of message targets of sRNA regulation, including sRNA conservation regions, structural accessibility of regions of sRNA and mRNA, and hybridization energy between the two RNAs	Bacteria	T
			RNAstructure (including ProbKnot, OligoWalk, bipartition, bifold, DuplexFold, Dynalign, Multilign, PARTS, TurboFold, etc.) (RNA–RNA) [259]	A software package for RNA secondary structure prediction and analysis, using both MFE and comparative approaches (more accurate than single sequence secondary structure prediction)	Accept MSA as input, the name of a sequence file (SEQ, FASTA) or structure file (CT, DBN) containing the input sequence	Constrained/Restrained structure prediction based on chemical mapping, enzymatic mapping, NMR, and SHAPE data Prediction of the accessible regions in an RNA target to oligonucleotide hybridization Calculation of thermodynamic features of sense–antisense hybridization Summary of tools: a) ProbKnot: Prediction of base-pairing probabilities, bimolecular structures with and without intramolecular structure b) OligoWalk: Equilibrium binding affinity of an oligonucleotide to a structured RNA target c) Bipartition: Partition function calculation for two interacting NA sequences without intramolecular pairs d) Bifold: Prediction of lowest free energy structure for two interacting sequences with or without intramolecular base pairs (DuplexFold) e) TurboFold: Calculation of conserved structures of more than 3 unaligned sequences using iteratively refined partition functions f) Dynalign, Multilign: Prediction of secondary structures common to two, unaligned sequences g) PARTS: Prediction of the common secondary structure, including base pair probabilities, for two unaligned sequences	All species	T
Conservation	Suboptimal Prediction	Local Interaction Length	OligoWalk (siRNA-mRNA) [137]	An online siRNA design tool using hybridization thermodynamics to predict efficient siRNA candidates for an mRNA sequence based on the statistical mechanics of the siRNA-target interaction with 78.6% efficient silencing	Only RNA oligomer is allowed, and 19 bases are recommended for siRNA design	Generation of a siRNA candidate table ranked by the probability of being efficient at knock-down. Prefilter score: The score calculated using Reynold et al. method [260] Generation of a thermodynamic table which includes: a) Net free energy change b) Free energy change of hybridised duplex between oligomer and target c) Melting temperature d) Free energy cost for opening base pairs in the region of complementarity to the target e) Free energy change of the self-structure of unimolecular oligo f) Free energy change of oligo-oligo dimer g) The number of suboptimal structures of the target used before and after the binding of oligomer h) Free energy difference between the 5′ and 3′ end of the antisense strand of siRNA, with windows of 2	All species	T
			RNAplex-aA and RNAplex-cA (RNA–RNA) [128]	A general RIP tool designed to quickly search possible hybridisation sites for a query RNA in large RNA databases as well as short interactions between two long RNAs	At least 1 FASTA file containing target and query RNA sequences or 2 CLUSTAL files as input	Computation of optimal and suboptimal structure (one structure per line) Conservation profile, consensus structure, and interactions with one, two and three types of base pairs Types of RNAplex: a) RNAplex-aA (accessibility and MSA as input) b) RNAplex-cA (interaction-only and MSA as input)	Virus, animal	T
No Conservation			RNAplex-a and RNAplex-c (RNA–RNA) [128]			c) RNAplex-a (accessibility) d) RNAplex-c (interaction-only)		
			RIsearch2 (RIsearch and GUUGle) (RNA–RNA) [140]	The first large-scale RIP tool using a seed-and-extend framework based on suffix arrays with a focus on perfect-complementary seed regions and extensions on both ends, applicable to all kinds of interaction predictions, and can be accessed via the conda package manager	RNA sequences in FASTA format	Quick localization of potential near-complementary interactions between given query and target sequences A modified Smith–Waterman-Gotoh algorithm based on di-nucleotides to approximate nearest-neighbour energy parameters Discovery of RRIs on genome/transcriptome-wide scale Parallel suffix array matching and seed extension Prediction of siRNA off-targets, including: a) Putative siRNA–RNA interactions b) Intersection with transcriptomic data c) Partition function d) Accessibility of binding sites e) Evaluation of siRNA off-target predictions and potential measurements f) Relationship between inhibition efficiency and off-targeting potential of siRNAs g) Validation of off-targeting potential measures	Multiple species	T
No Conservation	Suboptimal Prediction	Local Interaction Length	IntaRNA 2.0 (ncRNA-ncRNA) [134]	An upgraded version of IntaRNA, offers enhanced parameterization, flexible prediction modes and output formats, can be accessed via the conda package manager, integrated into the Galaxy workflow framework or ad hoc usage in the web interface	At least 1 FASTA file containing target and query ncRNA sequences	Visualization of new minimal energy profiles of RRIs Detailed investigation of interaction alternatives and detection of potential interaction site multiplicity Seed stability constraints Dangling end contributions	Bacteria	T
			IntaRNA (ncRNA-ncRNA) [135]	A program for fast and accurate RIP by incorporating seed constraints and interaction site accessibility, offers accurate sRNA binding site identification via optimal solution, prediction of optimal and suboptimal hybridisations (similar performance with RNAplex)	At least 1 set of noncoding RNA sequences in FASTA format with more than 1 but at most 100 sequences, each with a length ranging from 7 to 2000 nt	Prediction of the interactions in single organisms Summary of the best 100 predicted interactions Functional enrichment with region plots of top-25 predicted targets (an overview of the regions in the target and query sequences that play predominant roles) RRI output with ASCII chart Types of IntaRNA: a) IntaRNA- fast, heuristic RIP b) IntaRNAhelix- helix-based predictions c) IntaRNAexact- exact predictions like RNAup d) IntaRNAduplex- hybrid-only optimisation like RNAduplex e) IntaRNAsTar- optimised for sRNA-target prediction f) IntaRNAseed- identification of seed interactions only g) IntaRNAens- ensemble-based prediction and partition function computation	Bacteria	T
	No Suboptimal		InRNAs (RNA–RNA) [139]	A fast heuristic method to predict the specific (multiple) binding sites of two interacting RNAs, determine pairwise alignment, and handle complex joint structures	RNA pairs ranging from 20 to 60 nt	Prediction of the competitive RNA–RNA binding sites and RRIs Computation of the MFE joint secondary structure without pseudoknots, crossing interactions, and zigzags	All species	F
			BistaRNA (mRNA of ncRNA) [138]	A method for predicting multiple binding sites of target RNAs with reduced computational cost by binding profiles representing scores for hybridised structures	mRNA sequences of specific ncRNA	Prediction binding sites of target RNAs that are expected to interact with regulatory antisense RNAs Prediction of multiple binding sites of target RNAs Prediction of binding profiles that represent scores for hybridised structures Computation of accessible regions of the antisense RNA sequence	All species	T
			RNAup (RNA–RNA) [133]	A program that calculates the thermodynamics of RRIs by assessing the probability of a potential unpaired binding site, combining it with interaction energy to obtain the total binding energy, making it ideal for in-depth RIP especially when the interaction partners are known or when a candidate set has already been obtained by faster, less accurate methods	One (accessibility) or 2 (interaction) RNA sequences in FASTA format with a limit of 5000 nt per sequence	Two modes: a) Accessibility: identification of the region with the highest accessibility and its opening energy b) Interaction: Calculation of RRI between 2 RNA sequences, the best free energy of binding, its location, the optimal region of interaction, and its optimal structure (RNAduplex)	All species	T
No Conservation	No Suboptimal	Global Interaction Length	Sfold (siRNA and miRNA) [132, 261]	A software program developed to predict RNA secondary structures, assess mRNA and viral RNA target accessibility	RNA sequences in raw format, in FASTA format, or GenBank format (200 bases for an interactive job and 5000 bases for a batch job)	Two application modules: – STarMirDB: a database of precomputed transcriptome scale predictions – STarMir: miRNA binding site predictions for mRNA and target sequences) Outputs: – Prediction of target accessibility and rational design of antisense oligonucleotides or trans-cleaving ribozymes – Prediction of target accessibility and RNA duplex thermodynamics for rational siRNA design – Energetic characteristics of hybridisation between a structured target and a miRNA – Visualisation of comprehensive sequence, thermodynamic, and target structure features, a logistic probability as a measure of confidence for each predicted site, and a publication-quality diagram of the predicted miRNA–target hybrid	All species	T
	Suboptimal Prediction		RNApredator (RNA–RNA) [136]	A fast accessibility-based prediction of single sRNA targets via RNAplex with improved prediction specificity via the inclusion of accessibility and a database of over 2155 genomes and plasmids from 1183 bacterial species	A single small RNA sequence consisting of lower or uppercase letters (A, T, C, G, U), where T is automatically converted into U (with confirmed genome)	Computation of the full nonpseudoknot partition function of interacting strands in dilute solution Calculation of the concentrations, MFEs, and base-pairing probabilities of the ordered complexes below a certain complexity Computation of the partition function and base pairing of single strands including a class of pseudoknotted structures Prediction of the suboptimal interactions Design of ordered complexes Computation of putative target via RNAplex	Bacteria	T

A: Adenine; C: cytosine; G: guanine; lncRNA: long noncoding RNA; MFE: minimum free energy; miRNA: microRNA; mRNA: messenger RNA; MSA: multiple sequence alignment; NA: nucleic acid; ncRNA: noncoding RNA; NMR: nuclear magnetic resonance; nt: number of nucleotides; RIP: RNA–RNA interaction prediction; RRI: RNA–RNA interaction; RNA: ribonucleic acid; siRNA: short interfering RNA; srRNA: small regulatory RNA; sRNA: small RNA; T: thymine; TINCR: terminal differentiation-induced noncoding RNA; U: uracil

**Table 3:** Concatenation-based RIP tools based on MFE algorithms

Characteristic			Concatenation-based RIP Tool	Description	Input	Output	Applicable Species	Active (T)/ Inactive (F)	
Conservation	Suboptimal Prediction	Local Interaction Length	Nucleic Acid Package 4.0 (NUPACK 4.0) [148, 149]	• A growing software suite for the analysis and design of one or more species of interacting RNA strands. It enables analysis of nucleic acid sequences over complex and test tube ensembles containing arbitrary numbers of interacting strand species	At least 2 alignments of RNA sequences and allow specifications for the components, conditions of the RNA solution of interest, temperature, number of strand species, maximum complex size, strand sequences and strand concentrations	• Calculation of partition function, equilibrium base-pairing probabilities, MFE energy, proxy structure, suboptimal proxy structures, and Boltzmann sampled structures • Calculation of the partition function and MFE secondary structure for nonpseudoknot complexes of arbitrary numbers of interacting RNA strands • Calculation of the equilibrium concentrations for arbitrary species of complexes in a dilute solution • Calculation of equilibrium base-pairing observables for dilute solutions of interacting strand species via partition function and concentration information • Sequence design for >1 strand intended to adopt a nonpseudoknot target secondary structure at equilibrium	All species	T
No Conservation	No Suboptimal		UNAFold (Unified Nucleic Acid Folding and hybridization package) (DNAmelt & mFold) [146, 152]	• A tool with several closely related software applications available on the Worldwide Web for the prediction of the secondary structure of single-stranded nucleic acids; mFold has been replaced by UNAFold	One or 2 single-stranded RNA sequences in FASTA format with sequence name	• Prediction of RNA secondary structure (excluding pseudoknots) • Simulation of folding, hybridization, and melting pathways for one or two single-stranded NA sequences • Folding (secondary structure) prediction for single-stranded RNA via free energy minimisation, partition function calculations and stochastic sampling • Computation of entire melting profiles (plots), including melting temperatures (UV absorbance at 260 nm, heat capacity change (C(p)), and mole fractions of different molecular species	All species	T
	Suboptimal Prediction	Global Interaction Length	AccessFold (RNA–RNA, miRNA–mRNA, sRNA-mRNA) [262]	• A program for RIP with consideration for competing self-structure and allowing accessibility-based prediction as well as pairwise alignment	Two sequence files with sequence names for the first and second sequence	• Two approaches to evaluate accessibility: • Free energy density minimization • Pseudoenergy minimization • Minimization of the sum of free energy change and a pseudofree energy penalty for bimolecular pairing of nucleotides that are unlikely to be accessible for bimolecular structure • Prediction of binding sites that are split by unimolecular structures • Output is written to a CT file where the sequences are concatenated, with an intermolecular linker between them	All species	T
			PairFold [144]	• The first tool to predict suboptimal secondary structures of two interacting RNA strands and can handle complex joint structures	At least 2 sets of RNA sequences	• Prediction of the MFE pseudoknot-free secondary structure of two or more nucleic acid molecules via an extension of the Zuker and Stiegler algorithm [123] • Prediction of alternative low-energy suboptimal secondary structures for two NA molecules via suboptimal folding algorithm by Wuchty et al. [263] • Prediction of interactions between a probe and target RNA molecule or between pairs of strands in biomolecular nanostructures	All species	
No Conservation	Suboptimal Prediction	Global Interaction Length	MultiFold [144]	• The first program to handle multiple RNA strands	At least 2 RNA sequences and accept MSA as input	• Standard thermodynamic parameters of the Turner group prediction of the MFE pseudoknot-free secondary structure of two or more nucleic acid molecules • Prediction of alternative low-energy (suboptimal) secondary structures for two nucleic acid molecules	All species	
			RNAsoft (PairFold, CombFold, RNA designer, AveRNA, & HotKnots 2.0) (RNA–RNA) [143]	• A suite of RNA secondary structure prediction and design software tools, applicable for DNA sequences, and handles complex joint structures	Two RNA sequences with a description of a combinatorial set of RNA strands	• Summary of tools: • PairFold: Prediction of the MFE secondary structure formed by two input RNA molecules and interactions between a probe and target RNA molecule or between pairs of strands in biomolecular nanostructures • ComdFold: Prediction of the origin of a strand from a combinatorial set formed from RNA input strands and folding to a secondary structure with the lowest MFE • RNA designer: Designing an RNA sequence that folds to a given input secondary structure • AveRNA: Combination of the RNA secondary structures predicted by different algorithms to increase the overall accuracy • HotKnots 2.0: Prediction of short RNA secondary structures that are expected to form pseudoknots	All species	
	No Suboptimal		RNANUE (RNA–RNA) [147]	• A comprehensive and efficient analysis to detect RRIs from DDD (direct-duplex-detection) data	RNA sequencing files in a specific folder structure (the root folders must be specified for both treatment and control groups, and subfolders should represent arbitrary conditions that contain the read files)	• Split reads generation in SAM format • Clusters identification, including the IDs of the clusters, its length, size and genomic coordinates • Detection of RRIs, complementarity scores, and hybridization energies identification • MFE hybrid structure prediction and the probability in the ensemble of all possible interactions via RNAlib	All species	T
			RNAfold (RNA–RNA) [125]	• A web server which predicts secondary structures of single-stranded RNA sequences	RNA or DNA sequence in FASTA format with a limit of 7500 nt for partition function calculations and 10,000 nt for MFE-only predictions	• Interactive RNA secondary structure plot • RNA secondary structure plots with reliability annotation (partition function folding only) • Mountain plot (to predict and plot secondary structures)	All species	T
			RNAcofold (RNA–RNA) [125, 145]	• A program like RNAfold, but allows users to specify two RNA sequences that can form a dimer structure, capable of interaction-only MFE-based method, and can handle complex joint structures	RNA sequences are read from stdin in the usual format	• Calculation of secondary structures of two RNAs with dimerization • Computation of the hybrid structure of two molecules • Computation of MFE structures, partition function (pf) and base pairing probability matrix (using the −p switch) • Computation of equilibrium concentrations for all five monomers and (homo/hetero)-dimer species, given input concentrations for the monomers (since dimer formation is concentration dependent) • Generation of PostScript structure plots and "dot plot" files containing the pair probabilities	All species	T
			RNA–RNA interACTion prediction using Integer Programming (RactIP) (RNA–RNA) [163]	• A fast and accurate ML and probabilistic approach to predict RRI using integer programming, and handling complex joint structures	Two RNA sequences in FASTA format	• Integration of approximate information on an ensemble of equilibrium joint structures into the objective function of integer programming using posterior internal and external base-pairing probabilities • Prediction of RNA joint secondary structures under the general type of interaction including kissing hairpins • Prediction of the maximum expected accuracy (MEA) structure using integer programming (IP) with threshold cut via GNU Linear Programming Kit (GLPK)	All species	T

CT: continuous tone; DNA: deoxyribonucleic acid; GNU: Gnu's Not Unix; MFE: minimum free energy; MSA: multiple sequence alignment; NA: nucleic acid; nt: number of nucleotides; RIP: RNA–RNA interaction prediction; RRI: RNA–RNA interaction; RNA: ribonucleic acid; UV: ultraviolet

#### Interaction-only approach

The first RIP method is known as the ‘interaction-only (IO)’ approach because it only considers intermolecular base pairs during computation and in the final predicted outcome [87]. The MFE derived from intermolecular base pairs between two RNA strands is called the hybridization energy. IO possesses fast algorithmic speed but lower accuracy, as it neglects intramolecular RNA structures that might disrupt and constrain certain intermolecular interactions. IO prediction tools compute the overall Gibbs free energy (ΔG) and determine the direction of RNA folding. The stable RNA structure is determined by minimizing free energy using thermodynamic data such as temperature and chemical composition. The goal is to find the structure with the lowest Gibbs free energy, indicating its most stable conformation under the given thermodynamic conditions. Examples include DuplexFold [126], targetRNA [127], RNAhybrid [126], RNAplex [128], RNAduplex, RNAaliduplex [125], RIsearch [129] and GUUGle [130] (Table 1).

The DuplexFold server predicts the lowest hybrid free energy conformation of two RNA sequences based on intermolecular base-pairing, whereas targetRNA identifies base-pair complementarity and calculates RRI scores using the MFE model for RNA duplexes [127]. Following targetRNA, RNAhybrid predicts eukaryotic miRNA target and prokaryotic sRNA target interactions [126]. Both targetRNA and RNAhybrid heavily rely on the energies of stacked back-to-back base pairs, interior loops, and bulges for their prediction. For more efficient computation and less complexity, the consideration of long interior loops is limited and excluded during the RIP process. Conversely, database-based RNAplex is explicitly designed to search for potential hybridization sites in a query RNA. It implements a slightly different energy model than RNAhybrid, shortening computational time and enabling target search on highly stable interactions.

Both RNAduplex and RNAaliduplex, included in the Vienna RNA 2.0 package, predict conserved RRI between two alignments [125]. In contrast, the RIsearch algorithm is designed to rapidly scan genome-wide ncRNA–RNA pairs. It incorporates a simplified Turner energy model to the Smith–Waterman–Gotoh algorithm, approximating the Turner nearest-neighbour energy model using the dinucleotide scoring matrix [129]. Interestingly, GUUGle stands out by not calculating Gibbs free energies to determine optimal interactions. Instead, it generates all ungapped interactions over a user-specified length, serving as an absolute baseline for predicted performance. Moreover, GUUGle is designed to reduce the search space for more complex algorithms [130]. Overall, all the IO methods predicted RRI solely based on intermolecular base pairs.

#### Accessibility-based approach

To overcome the shortcomings of IO prediction tools, the accessibility-based (AB) approach was introduced to predict intra- and intermolecular base pairs [87]. AB uses the McCaskill partition function algorithm to predict the pairing likelihood of single nucleotide sequences at each position of the input sequence data [131]. The stability of intermolecular interactions at specific positions is determined by calculating stacking base pairs and the likelihood of intramolecular base pairs being inaccessible within the RNA molecules. The energy needed to prevent interacting RNA segments from forming intramolecular base pairs is known as accessibility energy. Sfold [132], RNAup [133], IntaRNA [134, 135], RNAplex [128], RNApredator web server [136] (updated version of RNAplex), OligoWalk [137], BistaRNA [138], inRNAs [139], RIsearch2 [140], RIblast [141] and targetRNA2 [142] are examples of prediction tools that adopted the AB approach (Table 2).

The Online Sfold tool predicts RNA secondary structure, target accessibility and hybridization energy [132]. It can compute the accessibility of binding regions and calculate the MFE of the RNA duplex via RNAup [133], IntaRNA 2.0 [134] and RNAplex [136]. However, RNAplex and RNAup cannot predict pseudoknots, while IntaRNA 2.0 is limited to interactions between single hairpin loops and excludes kissing hairpins (more complex pseudoknots/multiloops). OligoWalk predicts the hybridization of oligonucleotide binding by calculating the total free energy of an RNA sequence to the target sequence of a known structure [137]. BistaRNA and inRNAs provide insights into RNA accessibility and can predict multiple binding sites [138, 139]. Similarly, RNApredator is a fast accessibility-based prediction tool for single small RNA targets that uses a full nonpseudoknot partition function of interacting strands in a dilute solution [136].

RIsearch2 and RIblast are genome/transcriptome-wide scale RIP tools that implement the seed-and-extension approach to discover seed regions using suffix arrays and possess faster computational speed (64×) than other existing similar programs [141]. The seed regions are further refined using an energy model of the predicted RNA secondary structure [140]. On the other hand, TargetRNA2 is a tool for identifying targets of small regulatory RNAs (sRNAs) in bacteria via conserved regions, secondary structures, individual mRNA target secondary structures, and sRNA–mRNA hybridization energy. In RIP, TargetRNA2 suggests that the more conserved two sRNAs have in common, the more likely they are to interact with one another.

#### Concatenation-based approach

The third subclass of the MFE-based RIP tool involves both intermolecular and intramolecular base pairing of RNA. This approach is called concatenation-based, where two input sequences are concatenated and run through classical RSP algorithms to compute internal and external base pairs simultaneously [87]. Examples of concatenation-based tools include RNAsoft [143], PairFold [144], RNAfold [125], MultiFold [144], RNAcofold [125, 145], UNAFold (mfold/RNAfold) [146], RNAnue [147] and NUPACK [148, 149] (Table 3). However, they are limited due to the inability to predict pseudoknots accurately, where the base pairs are not well nested but overlap with each other.

In 2003, Andronescu *et al.* introduced an RNAsoft suite of programs to predict the secondary structure (PairFold), test combinatorial tag sets (CombFold) and design RNA strands (RNA Designer) [144, 150, 151]. PairFold is the first tool to predict suboptimal secondary structures of two interacting strands, and MultiFold is the first to handle multiple strands. Both programs use the standard thermodynamic parameters of Turner for RNA molecules [113, 132, 144]. RNAfold is a web tool that predicts the secondary structures of single-stranded RNA sequences [125]. Compared to RNAfold, RNAcofold allows the prediction of RNA secondary structures of single-stranded RNA sequences upon dimer formation [125, 145]. On the other hand, unified nucleic acid folding and hybridization package (UNAFold) is an amalgamation of mfold and DINAMelt. It predicts the pseudoknot-free RNA secondary structure of a single RNA sequence by simulating its folding, hybridization, and melting pathways. The prediction minimizes the global free energy using an improved algorithm by Zuker and Stiegler [125, 146, 151, 152]. RNAnue predicts inter- and intramolecular RRIs using complementary strands of double-stranded RNA information through direct-duplex-detection (DDD) methods [147].

#### Multiple sequence alignments and complex joint approach

Sequence alignment is a method to align DNA, RNA or protein sequences, predicting conserved regions that represent functional or evolutionary relationships between two sequences. Pairwise alignment determines the best-matching pattern of two sequences, whereas multiple sequence alignment involves multiple sequences simultaneously. Local alignment identifies local regions with the highest similarity level in sequences, whereas global alignment spans the entire sequence. RNAPLEX [128] and RNAduplex [125] are programmes that predict conserved RRIs using sequence alignments.

Another RIP tool of the MFE algorithm is known as the ‘complex joint’ (CJ), owing to MFE computation to identify the RRI between multiple RNA alignments. Unlike single RNA secondary structure-based RIP tools [33, 44], CJ can handle more complex joint structures with multiple interaction sites [153–158]. This capability is crucial, as ncRNAs often interact with target mRNAs in gene translation. Moreover, these relatively long regulatory antisense RNAs are not fully complementary to their target sequences. Instead, they rely on stable joint structures with mRNA via loop–loop interactions to facilitate regulatory functions [155]. Nevertheless, predicting these RNA secondary structure complexes with MSA is challenging (nondeterministic polynomial-time (NP)-hard problem), and only a few dedicated tools are available.

MultiRNAFold is a CJ-based package that includes three types of software: SimFold, PairFold and MultiFold [144]. It computes the MFE for predicting the secondary structure of interacting RNA molecules. Early attempts, such as PairFold [144] and RNAcofold [159], treated two interacting RNA sequences as a single sequence but faced challenges in predicting complex interactions such as kissing hairpins.

In 2007, Dirks *et al.* [160] introduced the NUPACK package, which efficiently computes the partition function of a single to multiple RNAs and concatenates input sequences in order, considering their symmetries and sequence heterogeneity. Similarly, BPPart, a revised algorithm of rip [157] and piRNA [154], computes the partition function for joint structures. The energy model is simplified by ignoring the entropy systems while retaining the thermodynamic information captured by more complex models [161]. The inRNAs algorithm predicts multiple binding sites in an RNA complex [139], while RIG utilizes multiple context-free grammars to model RRI [162]. Other CJ tools, such as IRIS [156], inteRNA [153] and piRNA [154], were previously available, but they are obsolete or no longer supported.

This review highlights that CJ methods are limited to relatively short RNA sequences to improve runtime performance. Although longer sequences cover a broader class of interacting RNA structures simultaneously, they are highly resource intensive and impractical for genome-wide scans. To overcome this challenge, Kato *et al.* [163] developed RactIP (RNA–RNA interaction prediction using integer programming), a novel method to increase the input RNA sequence length while optimizing runtime performance and prediction accuracy using the threshold cut technique.

### Comparative sequence analysis for RNA structures and RNA–RNA interaction prediction

The structures of functional ncRNAs are crucial in understanding their functions and evolutionary conservation. Structural alignment compares a folded RNA to known reference ncRNAs, identifying similar regions called ‘conserved regions.’ Comparative sequence analysis allows the identification of these conserved regions. The alignment score represents the similarity in the ncRNA sequence and structure. Comparative analysis suggests that RNA-forming base pairs in RNA secondary structures tend to be more conserved and covary during evolution to maintain Watson–Crick and wobble pairings (compensatory mutations) [87, 164, 165]. This supports the theory that base pairs with fully conserved or retained structures from compensatory mutations are more functionally important than unconserved base pairs [87].

Multiple sequence alignment (MSA) is one of the oldest comparative studies used to detect common secondary structures from a set of homologous sequences. By including well-aligned and sufficiently divergent homologues, MSA provides valuable information for predicting evolutionarily conserved base pairs. This approach also significantly improves the accuracy of the RSP tool and overcomes shortcomings of the MFE-based approach, such as the difficulty in aligning RNA sequences with low similarity (<60%) and folding different primary sequences into the same secondary structures.

**Figure 4:**
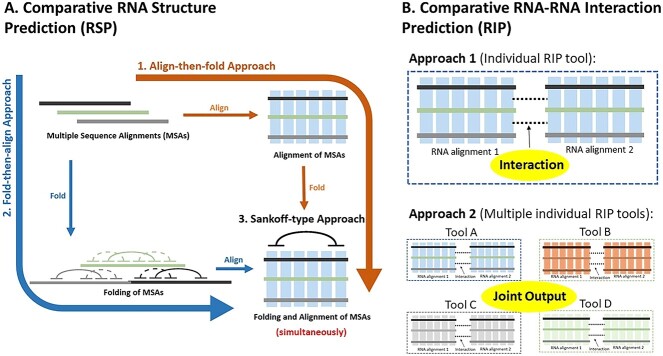
Comparative RNA structure prediction (RSP) and RNA–RNA interaction prediction (RIP). (**A**) The three main approaches in comparative RSP: 1. Align-then-fold approach; 2. Fold-then-align approach and 3. Sankoff-type approach (alignment and folding simultaneously); (**B**) The two main approaches in comparative RIP are (i) interaction between two alignments via an individual RIP tool and (ii) interactions obtained from the joint output of multiple individual RIP tools (adapted from [257]).

To date, comparative sequence analysis (homology) is more accurate than DPP approaches in RSP [166, 167]. This review highlights three major components of comparative sequence analysis (Figure 4**A**), including several examples of freely available homology-based tools in RIP, as tabulated in Tables 4–7 [164].

**Table 4:** Align-then-fold RSP tools based on comparative sequence analysis

Characteristic			Align-then-fold RSP Tool	Description	Input	Output	Applicable Species	Active (T)/Inactive (F)
Conservation	Suboptimal	Local Interaction Length	TurboFold II (part of RNAstructure) (RNA–RNA) [264]	An RNA structural alignment and secondary structure prediction informed by multiple RNA homologues, involving MFE	Homologous RNA sequences	Folding of a collection of RNA homologues via an iterative process instead of solving the joint problem of aligning and folding multiple RNA sequences Estimation of base pairing probabilities for each sequence and alignment posterior probabilities for each pair of sequences	All species	T
	No Suboptimal		RNAalifold (RNA–RNA) [172]	One of the oldest and most widely used tools for consensus structure prediction from RNA alignments, involving MFE	Multiple RNA alignments in CLUSTAL W and FASTA format	Computation of the MFE structure that is simultaneously formed by a set of aligned sequences Improved consensus RSP for RNA alignments Interactive RNA secondary structure plot RNA secondary structure plots with reliability annotation (partition function folding only) Mountain plot formation	Virus, bacteria, human	T
			Pfold [173]	An improved RNA secondary structure prediction software using the SCFG model, instead of using an explicit evolutionary model and a probabilistic model of structures	An alignment of up to 40 sequences and 500 positions in FASTA format with a phylogenetic tree relating the sequences	Calculation of structure posterior probability based on individual probabilities for alignment columns or pairs of columns in the case of a base-pair Estimation of the tree using a maximum likelihood approach in the SCFG model [265] Prediction of structure given as a bracket notation via CYK algorithm [266] Evaluation of the reliability of the prediction for each position A dot plot representing the overview of the prediction	Virus, bacteria, human	T
		Global interaction length	PETfold (RNA–RNA) [174]	A web server for intra- and intermolecular structures of multiple RNA sequences, involving concatenation MFE-based method	One MSA in FASTA format	Integration of both the thermodynamic and evolutionary paradigms into one model to predict: (a) Intra- and intermolecular RNA structures (b) Pairing reliability of base pairs	Bacteria, virus	T

CYK: Cocke–Younger–Kasami; MFE: minimum free energy; MSA: multiple sequence alignment; RNA: ribonucleic acid; RSP: RNA structure prediction; SCFG: stochastic context-free grammars

**Table 5:** Sankoff-type (sequence-based heuristics) RSP tools based on comparative sequence analysis

Characteristic			Sankoff-type (Sequence-based Heuristics) RSP Tool	Description	Input	Output	Applicable Species	Active (T)/Inactive (F)	
Conservation	Suboptimal Prediction	Local Interaction Length	Dynalign (part of RNAstructure) [267]	A web server for predicting common secondary structure in RNA homologues with domain insertions, including structural alignment and offering improved RSP accuracy by combining MFE and comparative RSP	Two RNA sequences (homologues)	Output a sequence alignment and a common structure for the two sequences. Prediction of the conserved pseudoknot-free secondary structure and the structural alignment of the sequences	Bacteria, virus, eukaryote	T
			Multilign [268]	An algorithm to predict secondary structures conserved in multiple RNA sequences, similar to Dynalign but with computational complexity that scales linearly in the number of sequences	At least two RNA sequence alignments	Prediction of the lowest free energy RNA secondary structure common to multiple sequences Improved prediction accuracy by keeping genuine base pairs and excluding competing false base pairs	Bacteria, virus, eukaryote	T
			MXSCARNA (multiplex stem candidate aligner for RNAs) [185]	A multiple alignment tool for RNA sequences using a progressive alignment approach based on the pairwise structural alignment algorithm of SCARNA, consuming less computational time and memory for large-scale analyses with better alignment accuracies	Two RNA sequences and accept MSA as input	Output a sequence alignment from a pair of RNA sequences based on the predicted common secondary structure Output from pairwise alignments to progressive multiple alignments with improved score functions, and simultaneously construct multiple alignments and the associated common secondary structures	Bacteria, virus, eukaryote	T
		Global interaction length	Stemloc [184]	A ML and probabilistic-based program for multiple alignment of RNA using SCFG, including structural alignment	Two RNA sequences (homologues), capable of pairwise alignment of multiple sequences	Output in Stockholm format, including the sequence names, the coordinates of matches, the alignment, the consensus primary sequence, the secondary structure of each sequence, the consensus secondary structure, and the log-odds score of the alignment in bits	Bacteria	T
	No Suboptimal	Local interaction length	CARNA (constraint-based alignment of RNA ensembles) [269]	A tool for multiple alignment of RNA molecules, involving MFE and predicting base pair probability for each RNA sequence with options for handling pseudoknots using RNAfold or without pseudoknots via NUPACK	A set of RNA sequences in FASTA format and one dot plot per sequence in PostScript format	Computation of optimal alignment of the sequences with respect to a sequence and structure similarity-based scoring Generation of conservation dot plots with the most likely base pairs of the consensus Representation diagram of sequence conservation and compatibility of base pair	Bacteria, eukaryote, virus	T
		Global interaction length	Foldalign version 2.5 (ncRNA) [182]	A new multithreaded version of Foldalign for pairwise structural RNA alignment, including structural alignment	Two RNA sequences or entire sequences with lengths up to 10,000 nt and a maximum alignment length of 1000 nt	Scanning for more than the best-scoring RNA structures Capable of discovery of ncRNAs Effective in local structural alignments of sequences with low similarity	Bacteria	T

MFE: minimum free energy; ncRNA: noncoding RNA; nt: number of nucleotides; RNA: ribonucleic acid; RSP: RNA structure prediction; SCFG: stochastic context-free grammars

**Table 6:** Sankoff-type (base-pair probabilities) RSP tools based on comparative sequence analysis

Characteristic		Sankoff-type (Base-pair) Probabilities) RSP Tool		Description	Input	Output	Applicable Species	Active (T)/Inactive (F)	
Conservation	Suboptimal Prediction	Local interaction length	PMcomp [186]	A method to compute pairwise and progressive multiple alignments from the direct comparison of base pairing probability matrices, including structural alignment	Two RNA sequences	Computation of base pairing probability matrices via McCaskill’s approach Extraction of the maximum-weight common secondary structure and an associated alignment via a simplified variant of Sankoff's algorithms	Bacteria, human, virus	F
		Global Interaction Length	FoldalignM (dependent on Vienna RNA package) (ncRNA) [188]	A multiple RNA structural alignment method, to a large extent based on the PMcomp program	Two or more RNA sequences or entire sequences and allow MSA as input	Capable of structural alignments for ncRNA Discovery of new ncRNAs Identification of the structure of novel ncRNAs Alignments improvement for known ncRNAs	Bacteria, human, virus	T
			Murlet [189]	A practical multiple alignment tool for structural RNA sequences. It implements an efficient scoring system that reduces the time and space requirements considerably without compromizing on the alignment quality	RNA sequences in FASTA format with a maximum length of 300 nt	Computation of the match probability matrix (align-ability of each position pair between sequences and the base pairing probability matrix) Scoring of RNA alignment using the Sankoff algorithm Prediction of the consensus secondary structure of the alignment via external programs Better accuracy in alignment and structure prediction than ClustalW, Stemloc and RNAcast	Eukaryote	T
	No Suboptimal		LocARNA (local alignment of RNA)/LocARNA -P [187, 252]	A fast and accurate comparison of RNAs with respect to their sequence and structure	RNA sequences in FASTA format (recommendation for the analysis of RNAs ≤60% sequence identity, where alignments based on only sequence similarity are unreliable)	Generation of a multiple alignment together with a consensus structure Extraction of putative RNA classes from genome-wide surveys for structured RNAs Robust against false positive predictions (e.g., contamination of the input data with unstructured or non-conserved sequences) LocARNA: folding via RNAfold or mfold; alignment via RIBOSUM-like similarity scoring and realistic gap cost LocARNA -P is more accurate boundary prediction and improved detection of structural RNAs than LocARNA	Virus, bacteria, plant	T
		Local interaction length	StrAl with PETcofold (ncRNA) [190]	A progressive alignment of ncRNA using base pairing probability vectors in quadratic time, where a scoring function is available for sequence similarity as well as up- and downstream pairing probability	A set of alignments with several sequences per alignment	Alignment of ncRNA based on a heuristic method with reduced sequence-structure alignment to a two-dimensional problem similar to standard MSA	Viruses, bacteria, eukaryote	F

MSA: multiple sequence alignment; ncRNA: noncoding RNA; nt: number of nucleotides; RNA: ribonucleic acid; RSP: RNA structural prediction

**Table 7:** Fold-then-align RSP tools based on comparative sequence analysis

Characteristic			Fold-then-align RSP Tool	Description	Input	Output	Applicable Species	Active (T)/Inactive (F)
Conservation	Suboptimal Prediction	Local Interaction Length	MARNA (surpassed by LOcARNA) [252, 253]	An MSA method that considers both primary sequence and secondary structure, and is based on pairwise comparison with edit operations on arcs and bases	RNA sequences in FASTA format (max 3 for RNAsubopt)	Prediction of the consensus sequence and structure Structure computation via MFE (RNAfold); structural shape (RNAshapes); structural ensemble (RNAsubopt) Computation speed faster than MASTR	Eukaryote	F
			planACstar (RNA–RNA) [196]	A tool for fine-tuning the folding process and structural RNA alignments in the twilight zone	A set of alignments with several sequences per alignment	Prediction of conserved RNA secondary structure and offer improvement in the twilight zone via a combination of several tools: ClustalW, RNAalifold, RNAfold, RNAforester, and RNAalifold	Mammal	T
			RNAspa (part of ViennaRNA package) (ncRNA) [270]	A shortest path approach for comparative prediction of the secondary structure of ncRNA molecules via a simple string Edit-Distance algorithm	A set of unaligned RNA sequences	Prediction of the secondary structure for a set of ncRNAs in linear time in the number of molecules Generation of graph, where the layer of vertices represents the suboptimal solutions	Virus, bacteria	T
			RNAcast (RNA consensus abstract shape technique) (ncRNA) [193]	An alternative to the Sankoff algorithm for multiple RNA structure prediction	At least 2 RNA sequences	Enumeration of the near-optimal abstract shape space Prediction of the consensus of an abstract shape common to all sequences Prediction of the thermodynamically best structure with the common shape for each sequence Prediction of the consensus structures of ten or more sequences at once	Virus	F
	No Suboptimal		RNA Sampler (ncRNA-RNA) [198]	A sampling-based algorithm for common secondary RSP and structural alignment via graph-theoretical approach, with no limitation on predicting pseudoknots; and provide refinement of alignment and folding process	Two RNA sequences	Prediction of common RNA secondary structures in multiple unaligned sequences Measurement of stem conservation by adopting the stem assembly idea from comRNA [271]; and combining both intrasequence base pairing and intersequence base alignment probabilities	Animal, eukaryote	T
Conservation	No Suboptimal	Global interaction length	MASTR (multiple alignment and structure prediction of ncRNAs) [197]	A tool to solve simultaneous structure prediction and MSA, while providing refinement of alignment and folding process	At least 2 RNA sequences in FASTA format	Prediction of the consensus structures Possibility to add structural constraints Computation speed faster than FoldalignM	Human, eukaryote	T
			LaRA 2 (ncRNA-RNA) [200, 272]	A parallel and vectorised program for sequence-structure alignment of RNA sequences and capable of handling arbitrary pseudoknots	At least 2 RNA sequences in FASTA format	Analysis of large sets of RNA secondary structures in a relatively short time, based on structural alignment Derivation of structural motifs (based on the produced alignments) to search in genomic databases	Bacteria, virus, eukaryote	T
			T-Coffee (tree-based consistency objective function for alignment evaluation) [195]	A web server for the RNA MSA using structural information and homology extension	RNA, DNA and protein alignments from any source in FASTA format	Combination of a collection of multiple or pairwise; global or local alignments into a single tool Estimation of the level of consistency/alignment accuracy of each position within the new alignment with the rest of the alignments Evaluation of RNA alignment and outputs a coloured version indicating the local reliability Evaluation of MSA using structural information with APDB and iRMSD Other types of T-coffee-related tools: a) M-Coffee- Alignment of RNA by combining the output of popular aligners b) R-Coffee- Alignment of RNA sequences using predicted secondary structures c) SARA-Coffee- Alignment of RNA sequences using tertiary structure	Parasite, bacteria, animal	T
	Suboptimal Prediction		CMfinder (ncRNA) [199]	A highly accurate covariance model-based RNA motif finding tool, derived from a small number of related sequences, to identify homologues in deeply diverged species	Unaligned RNA sequences	Prediction of RNA motif Inference of alignment and consensus secondary structure of an RNA Indication of the evidence of RNA secondary structure within the alignment. Summary of tools: RNAphylo: Assignment of a probabilistic score to an existing alignment, using an explicit phylogenetic model Hmmpair: Assignment of a score based on evidence of covariation that is supported by sequence conservation ScoreMotif.pl script: Combination of the previous two scores into one	Bacteria, archaea	T
			RNAforester (part of ViennaRNA package) [273]	A software comparing RNA secondary structures via forest alignment	RNA secondary structures from stdin or RNA sequences and structures in FASTA format	Calculation and comparing pairwise and multiple RNA secondary structure alignments via the tree alignment model Generation of alignments in ASCII format written to stdout Postscript drawings of structure alignments via option -2D	Bacteria, virus, eukaryote	T

2D: 2-dimensional; ASCII: American standard code for information interchange; DNA: deoxyribonucleic acid; MSA: multiple sequence alignment; ncRNA: noncoding RNA; RNA: ribonucleic acid; T-Coffee: tree-based consistency objective function for alignment evaluation

#### Align-then-fold approach

The align-then-fold approach extends RSP to multiple sequences by aligning them based on similarity and then predicting the structure with the lowest free energy that is shared by the largest number of sequences [168]. This approach requires a conventional alignment tool (e.g., ClustalW [169, 170], MAFFT [171]), followed by RSP tools (e.g., RNAalifold [172], Pfold [173]). The RNAalifold web server is one of the most important and commonly used tools (combined with score-based methods) [172], whereas Pfold includes compensatory mutations for accurate secondary RSPs [173]. Meanwhile, PETfold combines thermodynamic and evolutionary perspectives into a single model [174]. In short, the align-then-fold method is efficient for sequences with high similarity (>60%) and is a computationally less expensive method than the Sankoff-type and fold-then-align methods.

Table 4 summarizes a comprehensive overview of align-then-fold RSP tools.

#### Sankoff-type approach

The Sankoff algorithm is the most rigorous and computationally expensive approach to align RNA structure [175]. It combines structural prediction and sequence comparison simultaneously, ensuring similarity between structures by considering base-pair input in both [175–177]. This approach yields more accurate predictions than methods that separate folding and alignment steps, but it requires additional computer memory [178]. The Sankoff-based tools include MARNA [179], Foldalign [180–182], Dynalign [183], Stemloc [184] and MXSCARNA [185] (Table 5). They employ the Sankoff algorithm to explore the structural space and calculate the optimal secondary structure considering both sequence and structure conservation [175–177]. Additionally, some variants use sequence-based heuristics to reduce computational complexity and align efficiently.

Another approach uses McCaskill's algorithm to calculate base-pair probabilities via dynamic programming (Table 6), such as PMcomp [186] and LocARNA [187], whereas FoldalignM [188] and Murlet [189] employ a different algorithm called ‘maximum expected accuracy’ (MEA). StrAl with PETcofold [190] combines Sankoff and McCaskill’s algorithm, using Sankoff for RSP and McCaskill’s algorithm for base-pair probability calculation. This approach reduces the structural search space, computational complexity, and runtime by utilizing a simplified energy model based on precalculated base-pair probabilities from McCaskill’s algorithm, rather than directly calculating loop energies as in the Sankoff approach. Notably, RNA alignment and folding is not part of the Sankoff algorithm but a separate algorithm integrating sequence alignment and RSP, providing a comprehensive analysis of both sequence and structure aspects. It combines subsequence alignment quality-based heuristics and the simplified energy model of PMcomp to simultaneously align and fold unaligned RNA sequences [184, 191].

#### Fold-then-align approach

The fold-then-align method involves first predicting the secondary structures of RNA sequences and then identifying the structure with the lowest free energy across all sequences. This method often employs MSA to improve conserved RSPs. Another approach explores a middle path, where individual secondary structures are identified for each sequence in sets, followed by postprocessing to determine the optimal structure shared by all sequences. However, the accuracy depends on the quality of input RNA structures and may be limited by the number of matched homologous sequences, leading to potential false positives. Consequently, the overall alignment quality is typically affected by individual RSP approaches [192]. RNAforester [193], RNAcast [193] and aliFreeFoldMulti [194] are examples of applications implementing the fold-then-align method (Table 7).

To improve accuracy despite limitations in alignment quality, Notredame and colleagues developed the T-Coffee tool by implementing a preprocessing procedure that generates a library of local and global pairwise alignments [195]. It creates a consensus MSA by combining signals from diverse heterogeneous sources, such as sequence and structure alignment programs. Other methods, including planACstar [196], MASTS [197] and RNA Sampler [198], use sampling techniques to refine alignment and folding structures. However, CMfinder [199] and LaRA [200] stand apart from conventional categories because CMfinder specifically detects new ncRNA families by combining RSP and covariance models, whereas LaRA focuses on the identification of local RNA alignments considering both sequence and secondary structure conservation. In short, thermodynamic-based methods work with single RNA sequences due to similar algorithms as RSP systems, while comparative sequence analysis methods require MSA to enhance the accuracy and performance of RSP or RIP.

#### Pairwise alignments

The conventional approach for comparative sequence analysis mainly focuses on RSP due to several challenges in detecting RIP. For instance, the limitation of prediction within *in vitro* settings, the prevalence of false-positive predictions due to the high magnitude of predicted RNA–RNA duplexes and potential interaction partners, and the impact of external factors (other interacting RNAs/small ligands/proteins *in vivo*). Comparative RIP identifies the role of an RNA regulator via direct base-pairing with its target RNA.

Two primary strategies for comparative RIP are shown in Figure 4**B**. Similar to comparative RSP, the first RIP method (individual RIP) predicts the interaction between two alignments rather than two distinct sequences. Hypothetically, strong sequence signals distinguish binding sites and interactions based on their conserved structural residues. It is commonly believed that homology can help deduce binding sites and interactions. Tools such as PETcofold [174] and RNAripalign [201] leverage this hypothesis. PETcofold is an extended version of PETfold capable of predicting conserved RRIs [174], whereas RNAripalign identifies RRIs based on sequence and structural conservation [201].

Richter and Backofen [202] proposed that interaction sites between RNAs may not always be strictly conserved, suggesting that conserved interactions can occur even without precise conserved interaction sites. However, their statements contradict most of the alignment-based hypotheses that assume strict conservation of interaction sites. Henceforth, a new method combining individual RIP tools without requiring a strict consensus is introduced. It generates more reliable results and uncovers conserved regulatory mechanisms across different systems. This second method outperforms individual RIP tools. RNAhybrid, published by Krüger and Rehmsmeier in 2006 [203], predicts homologous miRNAs on orthologous targets from various organisms. However, duplex energies predicted by RNAhybrid must be transformed into *P* values, as the former is strongly influenced by the GC content and frequency of dinucleotides of the selected organisms. As duplex prediction relies on base-pair stacking, maintaining the dinucleotide frequency is crucial, and mononucleotide shuffling would prevent the generation of random sequences that accurately represent the features of the nonrandom system. The joint *P* value is used to identify possible interactions between two RNA alignments [25]. Similarly, CopraRNA uses Hartung's method to compute a joint *P* value for a cluster of homologous RNA sequences [204, 205].

Table 8 provides a comprehensive summary of RIP tools focussing on pairwise alignment in comparative sequence analysis.

**Table 8:** RIP tools based on pairwise alignment in comparative sequence analysis

Characteristic			Comparative RIP Tool	Description	Input	Output	Applicable Species	Active (T)/Inactive (F)	
Conservation	Suboptimal	Local Interaction Length	RNAripalign (part of rip) (RNA–RNA) [201]	A RIP tool based on MSA, using a priori folding algorithm implemented in C as part of the rip package (single sequence-pair folding algorithm)	Two given MSA (allow incorporation of structure constraints as input parameters)	Computation of the partition function Calculation of the base pairing probabilities and hybrid probabilities Prediction of a set of Boltzmann-sampled suboptimal structures consisting of canonical joint structures that are compatible with the alignments	Bacteria, virus, eukaryote	F
	No Suboptimal		CopraRNA (interaction calculated by IntaRNA) (sRNA-sRNA) [205, 274]	A tool for sRNA target prediction	At least 3 homologous sRNA sequences from 3 distinct organisms in FASTA format	Computation of whole genome predictions by a combination of distinct whole genome IntaRNA predictions A table with a sorted p value of target candidates for the entered homologous sRNAs Results of DAVID functional enrichment for the top 100 target candidates of CopraRNA and IntaRNA Calculation of interaction via IntaRNA Conservation of the sRNA/target interactions for the top 25 predicted targets Region plot (an overview of the regions in the target and sRNA sequences that play predominant roles in the statistically significant interactions)	Human, bacteria, virus	T
		Global interaction length	PETcofold (mRNA–RNA) [174]	A program predicting conserved interactions and structures of two RNA MSA, involving MFE, concatenation, and complex joint	Two MSA with at least three shared sequence identifiers in FASTA format	Prediction of intramolecular base-pair reliability Prediction of partial structure probability Identification of pairwise mRNA interaction site sequence Prediction of RNA joint secondary structures Prediction of intermolecular kissing hairpins	Bacteria, virus	T

MFE: minimum free energy; mRNA: messenger RNA; MSA: multiple sequence alignment; ncRNA: noncoding RNA; RNA: ribonucleic acid; sRNA: small RNA

### Pseudoknots: Loops and helical stems in RNA folding thermodynamics

RNAs contain an abundance of motifs, which are defined as discrete sequences or combinations of base juxtapositions. Structural motifs in RNA can form pseudoknots by base-pairing of single-stranded RNA regions in the hairpin loop with complementary nucleotides in the RNA chain [206]. The H-type pseudoknot is the most basic example, with a hairpin loop interacting with complementary nucleotides outside the loop [207]. Pseudoknots are critical components of RSP and RIP due to their involvement in translation readthrough mechanisms and are essential for identifying RNA complex functions [208]. Hinh *et al.* [209] also discovered a novel role of the ‘trans-pseudoknot’ RRI in the functional dimerization of human telomerase.

Additionally, the relationship between pseudoknots, RNA folding stability and conformational changes suggests that the interplay between loops and helical stems is essential in calculating RNA stability and folding thermodynamics [210–213]. Evaluating folding thermodynamics involves applying energy parameters to calculate the conformation energy and chain entropy, but this process can be computationally demanding and is limited to specific subclasses of pseudoknots [214].

For instance, using the DPP algorithm, Rivas and Eddy [215] developed an RSP tool called PKNOTS to fold optimal pseudoknotted RNAs (ranging from 100 to 200 nt), marking the beginning of prediction attempts on the secondary structure of RNA pseudoknots. PKNOTS can handle the broadest class of structures but is limited to small molecules due to its long running time [216]. Another DPP-based tool, HotKnots, offered faster prediction using a heuristic approach but could not guarantee the lowest free energy due to the vast conformational space and computational complexity. The search space is typically enormous, making an exhaustive search infeasible [216]. In short, existing DDP algorithms for pseudoknot prediction are both unreliable and inefficient.

Comparative methods are more reliable in predicting pseudoknot structures, but they are often selected in an ad hoc manner for specific purposes and require expert intervention [217]. The maximum weighted matching (MWM) algorithm can generate meaningful predictions, but it requires a large number of homologous sequences to detect strong covariance signals. However, the MWM algorithm is sensitive to noisy data such as misalignment, as it allows unrealistic interactions and may overlook the prevalence of helices as the most common structural elements in RNA structures [218, 219].

On the other hand, the iterated loop matching (ILM) algorithm combines both thermodynamic and comparative approaches to predict the secondary structure of RNA pseudoknots efficiently and reliably, even when only a few sequences are available. The ILM algorithm prioritises the formation of stable helices over computing a theoretically optimal structure, which proves to be beneficial by significantly enhancing the overall prediction accuracy. This advantage is particularly significant in situations where the available data are insufficient for a method such as MWM to generate reliable predictions using unrestricted models [220, 221].

Other examples of pseudoknot prediction tools are FlexStem and Kinefold. FlexStem constructed secondary RNA structures with pseudoknots by adding maximal stems based on the free energy model [222], whereas Kinefold used a long-term RNA folding simulation to predict pseudoknot structures with topological and geometrical constraints [223].

External pseudoknots or crossing interactions are formed when two interacting RNAs form pseudoknots. However, most of the thermodynamic-based tools disallowed the formation of pseudoknots and caused failure in predicting joint structures formed by nontrivial interactions between two RNAs. To address this problem, Eckart et al. developed NanoFolder, a program that predicts the base pairing of potential pseudoknots in RNA nanostructures. First, a simple energy model is used to calculate all possible helices, followed by a greedy algorithm to select the minimum free energy helices owing to their incorporation into the RNA complex [224]. Compared to NanoFolder, VfoldCPX uses a similar approach but a more advanced selection algorithm [225]. Meanwhile, IPknot could predict RNA secondary structures using a diverse set of pseudoknots from an individual sequence or MSA as an input [226]. Although comparative sequence analysis can predict pseudoknots, its accuracy is still limited. In brief, most of the computational methods predict the structure and RRI of pseudoknots using a thermodynamic-based approach, as reported in Table 9.

**Table 9:** RSP and RIP tools involving pseudoknots

Strategy	RSP and RSP tools involving pseudoknots	Description	Input	Output	Applicable Species	Active (T)/Inactive (F)
Thermodynamic-based approach	PknotsRG [275]	A web tool for folding and single sequence RNA secondary structure prediction including pseudoknots	A file containing one single RNA sequence in FASTA format	Folding and RSP, including pseudoknots, near-optimal structures and sliding windows Enumeration of suboptimal folding Visualization of RNA structure Alignment of RNA secondary structure Analysis of RNA secondary structure	Human, virus, bacteria	T
Thermodynamic-based approach	Kinefold (RNA–RNA) [223]	A web interface for RNA/DNA folding path and structure prediction including pseudoknots and knots	A string of unmodified RNA/DNA bases (limit of 400 bases for renaturation fold and cotranscriptional fold)	Folding kinetics of RNA/DNA sequences including pseudoknots and entangled helices Generation of a series of low free energy structures Generation of an online animated folding path Generation of a programmable trajectory plot focusing on a few helices of interest to each user	Virus, eukaryote	T
Thermodynamic-based approach	RNAMotif [276]	An RNA secondary structure definition and search algorithm including single strands, duplexes (antiparallel and parallel), pseudoknots, triplexes, and quadruplexes	A formal description of the permissible forms of the structure and the sequences contained within it	Description of RNA structural element, followed by the results of search in sequence databases, including the complete prokaryotic and eukaryotic genomes	Bacteria, virus	T
Thermodynamic-based approach	RCPred (RNA–RNA) [277]	A tool for secondary structure prediction of RNA complexes	Multiple RNA secondary structures in the complex with possible interactions in each RNA pairs	Prediction of internal and external pseudoknots, crossing interactions, and zigzags Generation of several suboptimal secondary structures	Bacteria, virus	T
Thermodynamic-based approach	Hyperfold (RNA–RNA) [278]	A web server for predicting NA complexes by interacting RNA strands with nonnested base pairings needed in silico secondary structure prediction	RNA and DNA strand sequences (including temperature and concentration)	Prediction of RNA multistrand structures, including RNA assemblies Prediction of structural information such as complex concentrations and base pairing Prediction of folding properties of RNA switches, RNA–DNA hybrid duplexes and RNA nanostructures resembling cubes and hexagons	Human	T
Thermodynamic-based approach	VfoldCPX (RNA–RNA) [225]	A web server for predicting RNA–RNA complex structure and stability	Two RNA sequences including temperature (recommendation: 300 nt for RNA secondary structures without crossing base pairs, ≤150 nt for structures with H-type pseudoknots, and ≤ 120 nt for RNA secondary structures with pseudoknots and hairpin-hairpin kissed structures)	Prediction of 2D RNA–RNA complex structures with at most one intermolecular crossing base pairing helix Prediction of RNA folding thermodynamics Prediction of RNA structure stability Prediction of kissing interactions in miRNA–target complex and assessment of miRNA activity	Eukaryote	T
Thermodynamic-based approach (statistical mechanics)	Vfold (ncRNA-RNA) [279]	A web server to predict RNA 2D, 3D structures and folding thermodynamics	RNA sequence in plain text form	Prediction of 2D structure (base pairs), via generation of RNA ensemble structures, including loop structure with different intraloop mismatches Prediction of 3D structure via motif scaffold assembly using structure templates from known PDB structures and refinement of structures through all-atom energy minimization Prediction of folding thermodynamics (heat capacity melting curve) and evaluation of free energies via experimental parameters for base stacks and loop entropy parameters	Human, virus	T
Thermodynamic-based approach (DDP heuristic algorithm)	HotKnots (RNA–RNA) [216]	A heuristic prediction of RNA secondary structures with or without pseudoknots	RNA sequences or sequence fragments	Identification of the lowest free energy structures at tree nodes via a standard free energy model Determination of tree pruning to explore alternatives from the most promising partial structures	Virus	T
Thermodynamic-based approach (DDP algorithm)	Pknots (RNA–RNA) [215]	An experimental code demonstrating a dynamic programming algorithm for RNA pseudoknot prediction	A single RNA sequence	Prediction of RNA structure with pseudoknots Prediction of the optimal minimum energy structure for a single RNA sequence Folding of optimal pseudoknotted RNAs ranging from 100 to 200 nt	Bacteria, virus	T
Thermodynamic-based approach (heuristic algorithm)	FlexStem (RNA–RNA) [222]	An algorithm improving predictions of RNA secondary structures with pseudoknots by reducing the search space	A ≥ 2 bp RNA secondary structure with a helical region or stem defined as an anti-parallel complementary strand	Simulation of the RNA folding process by successive addition of maximal stems Prediction of RNA structure with pseudoknots	Virus	T
Thermodynamic-based approach (empirical scoring function)	NanoFolder (RNA–RNA) [224]	A method for the prediction of the base pairing of potentially pseudoknotted multistrand RNA nanostructures	A set of RNA sequences combined with a descriptor for the desired target secondary structure	Prediction of the base pairing of potentially pseudoknotted multistrand RNA nanostructures Prediction of RNA complexes with nonnested base pairings; better performance than NUPACK, RNAcofold and PairFold Design of RNA sequence	Bacteria, human	T
Thermodynamic- or comparative-based approach (heuristic algorithm)	Iterated loop matching algorithm (RNA–RNA) [220]	An iterated loop matching approach to predict RNA secondary structures with pseudoknots	RNA homologous sequences	Identification of base-pairs for short sequences Prediction of pseudoknots with high accuracy on individual sequences Higher sensitivity and specificity than the maximum weighted matching method [219]	Eukaryote	T
Thermodynamic- or comparative-based approach	ProbKnot (part of RNAstructure) (RNA–RNA) [280]	A fast prediction of RNA secondary structure including pseudoknots	A sequence file of DNA or RNA	Prediction of the presence of pseudoknots in its folded configuration Visualization of pseudoknot in a circular structure Better performance than ILM, pknotsRG and HotKnots	Human, virus	T
Comparative-based approach	IPknot (RNA–RNA) [226]	A fast and accurate prediction of RNA secondary structures with pseudoknots using integer programming	A single sequence of RNA or MSA	Prediction of the MEA structure using IP with threshold cut and the consensus secondary structure with pseudoknots given an MSA input Decomposition of a pseudoknotted structure into a set of pseudoknot-free substructures Prediction of a base-pairing probability distribution that considers pseudoknots via a heuristic algorithm for refinement	Virus, eukaryote	T

2D: two-dimensional; 3D three-dimensional; bp: base pair; DNA: deoxyribonucleic acid; IP: integer programming; ILM: iterated loop matching; MEA: maximum expected accuracy; miRNA: microRNA; MSA: multiple sequence alignment; NA: nucleic acid; ncRNA: noncoding RNA; nt: number of nucleotides; RNA: ribonucleic acid

**Table 10:** Artificial intelligence-based RIP and RSP tools

Strategy	AI-based RIP and RSP tool	Description	Input	Output	Applicable Species	Active (T)/Inactive (F)
N-gram statistics language model	RIscoper (RNA Interactome Scoper) (RNA–RNA) [238]	The first tool for full-scale RNA interactome scanning via extraction of RRIs from the literature based on the N-gram model	Full texts or abstracts, with an online search tool connected to PubMed	Structured data of the extracted interactions in a machine-readable format such as interacting RNA partners, interaction types, contextual information, and metadata	All species	T
Score scheme (free energy parameter-refining approach based on ML)	Constraint generation- RNAsoft [241]	The first computational approach to RNA free energy parameter estimation that can be efficiently trained on large sets of structural as well as thermodynamic data	RNA sequence, all on one line; and RNA secondary structure in dot-parentheses format, all on one line	Computation of the energy values as the solution to a constrained optimization problem, followed by an update on the optimisation function to better optimise the energy parameters	All species	T
Score scheme (weighed approach based on ML)	ContextFold [243]	An RNA secondary structure prediction tool that applies feature-rich scoring models, whose parameters are obtained after training on comprehensive datasets	One or more RNA sequences in FASTA format and accept MSA as input with optional structure constraints	Prediction of RNA secondary structures, including base pairs, loops, and stems Assignment of confidence score alongside prediction for quality assessment Energy parameters associated with the predicted structure Visual representations of the predicted structure	All species	T
Score scheme (a probabilistic approach based on ML)	Stochastic context-free grammars [168, 281, 282]	An alternative probabilistic methodology for modelling RNA secondary structure prediction based on the success of Hidden Markov Models in protein and gene modelling	An alignment of RNA sequences	Prediction of RNA secondary structure	N/A	N/A
Predicting process based on ML (end-to-end approach)	SPOT-RNA [246]	An RNA secondary structure prediction web tool using an ensemble of 2D deep neural networks and transfer learning	Single RNA sequence or batch of sequences	Prediction of RNA secondary structure Calculation of base-pair probability of predicted secondary structure, which is useful for plotting PR-curve and checking the confidence of predicted base-pair Generation of 2D plots via the VARNA tool [283]	Human	T
Predicting process based on ML (hybrid)	Deep learning method for state inference [284]	An improved RNA secondary structure prediction using state inference with deep recurrent neural networks	Dataset of known input–output pairs	Prediction of states for RNA secondary structure via a deep bidirectional LSTM model Generation of synthetic SHAPE data Prediction of RSP using the NNTM model, incorporating the predicted states and synthetic SHAPE data	Bacteria, animal, eukaryote, archaea	T
Predicting process based on ML (hybrid)	DMfold [247]	A method to predict RNA secondary structure with pseudoknots based on deep learning and improved base pair maximization principle	Target RNA sequences with dot-bracket sequences as labels	Prediction of RNA secondary structure with pseudoknots	All species	T
Predicting process based on ML (hybrid)	MINT [248]	An automatic tool for analysing 3D structures of RNA and DNA molecules, their full-atom molecular dynamics trajectories or other conformation sets	A simple text file with a detailed description of the RNA or DNA structure in each conformation frame	Determination of the hydrogen bonding network resolving the base pairing patterns for each RNA conformation Identification of secondary structure motifs (helices, junctions, loops, etc.), pseudoknots and short-range interactions in trajectories of NA Analysis of RNA/DNA 3D structure and their full-atom molecular dynamics trajectories or other conformation sets (e.g., X-ray or NMR-derived structures) Estimation of the energy of stacking and phosphate anion-base interactions, including the energetic features and their evolution.	All species	T
Predicting process based on ML (hybrid)	CONTRAfold (CONditional TRAining for RNA Secondary Structure Prediction) (RNA–RNA) [242]	A secondary structure prediction method based on conditional log-linear models, a flexible class of probabilistic models which generalise upon SCFGs by using discriminative training and feature-rich scoring	Single RNA sequence	Prediction of the best RNA structure Calculation of base-pair probability of predicted secondary structure	All species	T

2D: two-dimensional; 3D: 3-dimensional; AI: artificial intelligence; DNA: deoxyribonucleic acid; LSTM: long short-term memory; ML: machine learning; NA: nucleic acid; NMR: nuclear magnetic resonance; NNTM: nearest neighbor thermodynamic model; RIP: RNA–RNA interaction prediction; RNA: ribonucleic acid; RRI: RNA–RNA interaction; SCFG: stochastic context-free grammar; SHAPE: selective 2′-hydroxyl acylation analysed by primer extension

**Table 11:** Advantages and shortcomings of MFE-based RIP and RSP tools

Type of RIP and RSP Tools	Advantages	Shortcomings	Ref.
Nussinov algorithm	The first DDP algorithm Efficient prediction of RNA molecule's optimal folding state through maximum base pairings calculation Show pattern of primary RNA structure Similar algorithmic structure as Zuker (energy minimization) Prediction of (restricted) crossing structure can be seen as an extension	No stacking of base-pairing considered Loop sizes not distinguished No special scoring of multiloops Inability to predict pseudoknotted helices Prediction of only one structure Not applicable to secondary RNA structures No suboptimal solutions Destabilization of multibranch loops/helical junctions Discontinuity in the formed base-pairs Low prediction accuracy High false positive base-pairs prediction	[114, 119–123, 285]
Interaction-only	Fast algorithmic speed Incorporating conservation data enhances specificity, leading to improved overall MCC performance A detailed view of RRIs	Lower accuracy Only consider intermolecular base-pairs during computation and in the final predicted outcome Heavy reliance on the energies of stacked back-to-back base-pairs, interior loops, and bulges for RIP Long interior loops are limited or excluded	[87, 127, 142, 203]
Accessibility-based	Prediction of both intra- and intermolecular base-pairs Suitable for all types of RRIs Compatible with eukaryotic and bacterial datasets Computation of RNA accessibility Prediction of multiple binding sites Ability to differentiate native interactions from a background in the bacterial dataset Ideal for *de novo* predictions, especially those with smaller run-times such as IntaRNA and RNAplex	Inclusion of MSA might decrease performance due to alignments of questionable quality The number of variables (such as alignment, percent of identity threshold, and suboptimal results settings) make it impractical in a *de novo* setting	[86, 138, 139]
Concatenation-based	Prediction of both intra- and intermolecular base-pairs Prediction of RNA secondary structures of single-stranded RNA sequences upon dimer formation Capable of handling multiple RNA strands	Challenge in predicting accurate pseudoknots Often computationally demanding, especially for large RNA molecules	[87, 143]

MCC: Matthews correlation coefficient; MSA: multiple sequence alignment; RIP: RNA–RNA interaction prediction; RNA: ribonucleic acid; RRI: RNA–RNA interaction; RSP: RNA structure prediction

## CHALLENGES IN RNA STRUCTURE AND RNA–RNA INTERACTION PREDICTION

With the rapid growth of biological data and technologies, there has been a surge in research for predicting structural RNA and RRI using computational approaches. However, researchers often overlook that the outputs from these tools do not reflect the actual RNA structure but rather assumption-based algorithms. In thermodynamic-based approaches, base pairs with higher free energies are occasionally ignored due to the lack of evidence in the literature. Representation of the ‘prediction/theoretical’ as the ‘true/actual’ RNA secondary structure or RRI results in the acceptance of an untested possibility without further investigation [82]. Moreover, the kinetic RNA structures that form during folding may serve as a crucial indicator of RNA functions [227]. For instance, riboswitches usually regulate metabolic functions via structural conformation instead of retaining a static native structure [228]. In addition, noncanonical base pairs also play a crucial role in forming tertiary RNA structures, necessitating their inclusion in the prediction process. Nevertheless, predicting both canonical and noncanonical base pairs remains a challenge. Noncanonical interactions must still be optimised as they may contain additional chemical probing information that facilitates RNA structure modelling and comprehension of functional RNA modules. In addition, predictions of RNA tertiary structure are less accurate in loop regions, where noncanonical pairs are required to evaluate structural details [229, 230].

Comparative-based techniques are limited by the need for a more extensive set of homologous sequences. Due to the limited knowledge of known RNA families, obtaining homologous sequences for all RNAs is unfeasible, resulting in a preference for score-based RSP with a single RNA sequence as input. The ‘predicted’ outputs should not be regarded as a substitute for comprehensive experimental RSP and RIP determination, as these algorithm-based prediction tools operate under the assumption that the nucleotides are likely to engage in secondary structure elements with the maximum predicted number of Watson-Crick base-pairings [117, 231, 232]. The automatic modelling methodology is another challenge in RSP and RIP tools. Due to limited experimental data, most currently available automated web servers only rely on RNA sequences as input with low accuracy. Therefore, integrating the experimental data into computational methods will be of assistance in enhancing the accuracy of RSP accuracy [79].

To improve the prediction accuracy of RIP and RSP tools, we concluded that five main challenges must be addressed as follows: (i) the limited number of examples with mapped interactions, (ii) limited focus on the kinetic RNA structures, (iii) the low specificity due to the restriction of single sequences, (iv) overreliance to ‘predicted’ output rather than experimental data and (v) the high cost for a search of complex types interactions provided a guaranteed maximum score is to be obtained.

## ARTIFICIAL INTELLIGENCE: CURRENT TRENDS AND FUTURE DIRECTIONS

Artificial intelligence has emerged as a powerful approach to predicting RNA structure and function [233]. In previous years, numerous prediction methods have been developed with the primary goal of identifying RNA structures that are likely to exhibit an MFE state, such as proteins [234]. However, over the past two decades, machine learning (ML) has been proposed as an alternative methodology to enhance the accuracy and calculation speed of RIP and RSP tools [235]. It was previously overlooked due to limited accuracy resulting from small training datasets and the constraints of simplistic ML models [236]. Due to the recent surge in RNA sequence data and advancements in ML, particularly deep learning (DL), the latest ML-based approaches surpass existing traditional methods in both accuracy and applicability, providing an advantage in tackling complex questions in structural biology while dealing with large datasets. DL algorithms leverage reference structures to train scoring parameters for decomposed substructure analysis, making them a more efficient and scalable alternative to traditional experimental procedures [237].

RNA Interactome Scoper (RIscoper) is a ground-breaking AI tool based on natural language processing (NLP) that extracts RNA structure and interactions from published literature using an N-gram model [238]. NLP automates tasks by extracting useful information from unstructured text and converting it into a structured format for computational analysis. NLP techniques have substantially improved in recent years, demonstrating their effectiveness across various domains. These include literature-based discovery, aiding the analysis of high-throughput data such as gene expression and genome-wide association studies [239]. ML-based approaches, on the other hand, can be categorised into two major groups, each aligned with a distinct phase in the RSP and RIP process: ML-based scoring schemes and ML-driven prediction processes.

Score-based methods are the most widely used traditional computational methods and have dominated the field of RIP and RSP. Scoring methods assume that RNA structures must satisfy specific score-based criteria, which can vary depending on the RNA folding mechanism, making secondary RSP an optimization problem. Dynamic programming (DP) algorithms are commonly employed to discover the optimal structure by dividing it into smaller components with individual scores and require a sophisticated scoring scheme with numerous parameters. However, DP algorithms are often deemed inefficient for large inputs, as their running time increases rapidly with the input size based on RNA sequence length and may overlook unique base pairs and weak interactions [233]. Understanding the RNA folding mechanism through the score-based method is thus a formidable challenge, in contrast to data-driven ML methods that do not rely on such mechanisms.

In this review, we highlighted two categories of ML-based methods for RIP and RSP according to the subprocess, e.g. (i) score scheme based on ML (free energy parameter-refining approach, weighted approach, and probabilistic approach) and (ii) ML-driven prediction process (end-to-end approach and hybrid approach) (Table 10). All ML methods within these two categories trained their models through supervised learning, wherein model parameters were adjusted based on input–output pairs. RIP and RSP primarily employ features such as free energy parameters, RNA sequences, and sequence patterns as input, and the trained model outputs can be either classification labels or free energy values. The probabilistic approach based on ML is one of the earliest scoring schemes that used stochastic context-free grammars (SCFGs) to predict RNA structures and interactions. Datasets containing RNA sequences annotated with known secondary structures are used to estimate the probability parameters of the SCFG model [240].

Andronescu and colleagues introduced the constraint generation (CG) method, a pioneering computational approach for estimating RNA-free energy parameters. This approach was designed to train on large datasets containing structural and thermodynamic information efficiently. By incorporating ML techniques, CG can predict and design RNA secondary structures with high accuracy [241]. Another notable tool, CONTRAfold, takes a different approach by using conditional log-linear models that generalise SCFGs through discriminative training and feature-rich scoring. This allows CONTRAfold to accurately predict RNA secondary structures based on probabilistic models [242]. ContextFold employs feature-rich scoring models that are trained extensively on large datasets [243]. This approach captures more complex relationships in the data, but there is a potential risk of overfitting, where the model becomes too specific to the training data and performs poorly on new, unseen data [244].

The ML-driven prediction process, on the other hand, adopts deep learning (DL) in predicting RNA structure [245]. SPOT-RNA, for instance, focuses on leveraging deep neural network learning to predict all base pairs, regardless of their association with local or nonlocal interactions. This approach leverages the power of DL to capture intricate patterns and features within RNA sequences [246]. To overcome limitations and enhance prediction accuracy, hybrid approaches have been introduced [233]. One example is the combination of thermodynamic and ML-based strategies, where the model of CONTRAfold and MFE (concatenation-based method and complex joint category) is used to predict RNA interactions [163, 242]. This hybrid method leverages the strengths of both thermodynamic principles and ML techniques to improve the accuracy of RIP. Nucleic Acid Package 4.0 (NUPACK 4.0), a hybrid tool, integrates ML-based and concatenation-based MFE methods for analysing and designing interacting RNA strands across multiple species. It enables the examination of RNA sequences in complex and test tube ensembles containing an arbitrary number of interacting strand species [148, 149].

For RSP, a method called DMfold has been proposed. DMfold combines deep learning and an improved base-pair maximization principle to predict RNA secondary structures with pseudoknots. By learning from similar RNA sequences instead of highly homogeneous sequences, DMfold reduces the requirement for auxiliary sequences and improves folding accuracy [247]. Motif identifier for nucleic acids trajectory (MINT) is an automatic tool to analyse 3D structures of RNA molecules, their molecular dynamics trajectories and other conformation changes [248]. On the other hand, CompaRNA utilizes a combination of 28 single-sequence methods and 13 comparative methods for continuous automated benchmarking [249, 250]. Although CompaRNA is primarily based on comparative sequence analysis rather than the ML method, it incorporates several ML-based tools, such as ContextFold and CONTRAfold, as part of its analysis pipeline [242, 243]. This demonstrates the synergy between comparative sequence analysis and machine learning, where ML algorithms complement evolutionary information and sequence conservation to improve predictions.

While ML techniques have significantly enhanced prediction methods in terms of accuracy, applicability, and processing speed, there remains a need for more sophisticated ML models to fully address the challenges of the RSP and RIP problems, particularly in predicting high-resolution structures [233]. Nevertheless, given the rapid expansion of RNA sequence data, the availability of high-performance hardware and continuous advancements in machine learning methods, there is a potential for the future development of cutting-edge RSP and RIP tools that could surpass traditional approaches in terms of both execution speed and accuracy.

## SELECTING THE BEST APPROACH: PRACTICAL RECOMMENDATIONS

Choosing the most suitable method for RIP or RSP depends on the specific research objectives. For instance, if the primary goal is on RIP and identifying binding sites, the IO method may be the preferred option since it excels at detecting interaction regions and base-pairing sites. However, IO methods are not designed to provide detailed structural information about the individual molecules involved [87, 127, 142, 203]. On the other hand, the concatenation-based method is selected for predicting the MFE structure of an entire RNA molecule, considering potential intramolecular interactions and structural elements. These methods offer a comprehensive perspective on the folding behaviour of RNA and have the capability to capture complex structures and interactions. However, they are frequently computationally demanding, particularly when applied to large RNA molecules [87, 143].

Accessibility-based MFE algorithms, as employed in RNAup, IntaRNA, and RNAplex, have demonstrated superior performance in RSP and RIP when compared to the previous two types of tools [128, 133, 134]. In an analysis of a bacterial dataset by Umu and Gardner in 2017 [86], these algorithms showed their ability to distinguish nearly half of the native interactions from the background noise. This accomplishment is facilitated by the integration of well-designed negative controls such as dinucleotide shuffling, enabling the utilization of predicted MFE values and distinct scoring mechanisms to effectively discriminate native interactions from spurious ones [86, 251]. These accessibility algorithms are especially valuable for *de novo* predictions, particularly in scenarios where computational efficiency is essential, as is the case with IntaRNA and RNAplex, given that candidate target RNAs can be extensive, spanning thousands of nucleotides [128, 134, 135, 205]. RNAplex, in particular, excels at identifying correct interaction regions that might be embedded within larger RNA targets [128]. In essence, accessibility-based MFE algorithms excel IO and concatenation-based tools due to their consideration of RNA sequence structural accessibility and evaluation of base-pairing potential, improving the capability to discern real interactions from nonspecific interactions.

In the context of selecting RSP and RIP tools based on comparative sequence analysis, Pfold and RNAalifold generally exhibit strong performance, especially for well-aligned short sequences [172, 173]. However, it is worth noting that RNAalifold outperforms in terms of speed and is better suited for well-aligned, longer RNA sequences [172]. For datasets comprising short sequences (< 200 bases) with significant diversity, Dynalign is a suitable choice because it does not rely on sequence similarity, and its scoring function excludes sequence comparisons [183]. In other scenarios, a combination of RNAalifold and/or Pfold can be employed to fold similar RNA sequences [172, 173], while RNAforester and/or MARNA can be used to align these folded RNA molecules [252, 253]. Notably, most of the MSA algorithms do not favour transitions over transversions or employ ad hoc two-parameter methods to model these distinctions (e.g. ClustalW [170]). This can be relevant because structural RNA sequences often evolve rapidly through structure-neutral mutations, which tend to involve transitions rather than transversions [254, 255]. Therefore, multiple sequence algorithms that utilise more sophisticated yet accurate models of sequence evolution are likely to produce improved alignments for folding [164].

**Figure 5:**
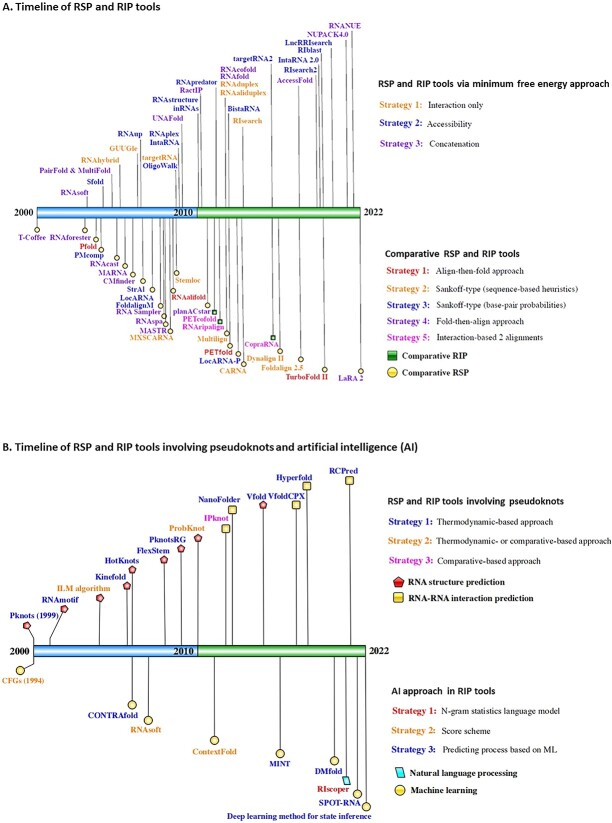
Timeline of RNA structure prediction (RSP) and RNA–RNA interaction prediction (RIP) tools. (**A**) Chronological overview of RSP and RIP tools, highlighting the different approaches via minimum free energy and comparative sequence analysis; (**B**) tools involving pseudoknots and artificial intelligence.

Table 11 offers a comprehensive overview of the advantages and limitations associated with MFE-based RSP and RIP tools. Additionally, Figure 5 presents a chronological depiction of the development timeline of RSP and RIP tools. Understanding this timeline is crucial for selecting the most appropriate tools based on research objectives and the evolution of available technologies.

## CONCLUSION

In recent years, the intersection of structure-based RNA analysis and computational biology has garnered significant attention as researchers recognize the crucial role of RNA structures in RNA function. Despite the availability of large-scale RNA sequence data, the development of computational algorithms for RSP and RIP has faced challenges, including the complexity of RNA structures and limited training datasets. These challenges have been met with advancements in computational techniques, and the progress in RSP tools has provided a solid foundation for the development of RIP tools, enabling a deeper exploration of the intricate network of RRIs and their functional implications. This review aimed to provide a comprehensive overview of existing computational tools for both RSP and RIP, focusing on two main types of RRIs and the strategies employed to predict them. ML has also been integrated into RIP and RSP methodologies. However, it is important to note that ML-based methods cannot yet replace wet lab experiments and traditional computational approaches to obtain high-resolution RNA structures or accurate RIP. Nonetheless, the advent of deep learning technologies and high-performance hardware will foster a new generation of RIP and RSP tools with improved accuracy and running speed.

**Key Points**

- Bridging the Gap: This comprehensive review features the connections between RSP and RIP, underscores the importance of RNA homologues, delves into the intricacies of pseudoknots and dissects the thermodynamics of RNA folding.
- Informative Figures: Our review includes figures that elucidate RRI types, emphasise the two core strategies within RIP, simplify explanations of each strategy subtype, and present chronological timelines that trace the evolution of RSP and RIP tools.
- Comprehensive Summary: A comprehensive summary of RSP and RIP tools, meticulously organised into detailed tables for each strategy type, is available. These tables encompass characteristics of the RSP and RIP tools, citations, concise definitions and functions, input and output specifications, applicable species, and status (active or inactive) for enhanced clarity.
- Challenges and Future Directions: We highlight five primary challenges in RSP and RIP and elaborate on how the integration of artificial intelligence through machine learning and deep learning holds the potential to significantly enhance RSP and RIP.
- Practical Recommendations: A dedicated section is included to offer valuable advice for the effective utilisation of RSP and RIP tools in various research applications.

## Data Availability

Not applicable.

## Author Biographies

**Francis Yew Fu Tieng** recently graduated from the Universiti Kebangsaan Malaysia. His expertise lies in cancer genomics and transcriptomics, focusing on single-cell RNA-sequencing on circulating tumour cells. Additionally, he studies the interplay of RNA–RNA interactions, and identify the potential of natural products in alternative cancer treatment.

**Muhammad-Redha Abdullah-Zawawi** is a research fellow at the UKM Medical Molecular Biology Institute (UMBI), Universiti Kebangsaan Malaysia. His expertise includes bioinformatics and computational systems biology.

**Nur Alyaa Afifah Md Shahri** is a research officer at the UKM Medical Molecular Biology Institute (UMBI), Universiti Kebangsaan Malaysia. Her research focuses on exploring the significance of bioinformatics in molecular biology research and cancer genomics research.

**Zeti-Azura Mohamed-Hussein** leads the Bioinformatics & Computational Systems Biology (BCSB) group at Universiti Kebangsaan Malaysia. Her expertise includes bioinformatics approaches like sequence- and structure-based analysis, integrative omics analysis, comparative genomics, network analysis, and genomic variation studies.

**Learn-Han Lee** leads the Sunway Microbiomics Centre at Sunway University, Malaysia. He perceives teaching as a significant responsibility that enables him to fulfil his role as a scientist specializing in biochemistry, molecular biology, microbiology, and immunology.

**Nurul-Syakima Ab Mutalib** is a senior research fellow at the UKM Medical Molecular Biology Institute (UMBI), Universiti Kebangsaan Malaysia. Her expertise lies in cancer genomics, transcriptomics, and epigenomics, focusing on non-coding RNA as cancer biomarkers (microRNA). Additionally, she studies genetic variants in sudden cardiac death and identifies novel actinomycetes for potential antibacterial and anticancer drug discovery.

## References

[1] Watson JD, Crick FH. Molecular structure of nucleic acids; a structure for deoxyribose nucleic acid. Nature 1953;171:737–8. 13054692 10.1038/171737a0

[2] Crick FH . The origin of the genetic code. J Mol Biol 1968;38:367–79. 4887876 10.1016/0022-2836(68)90392-6

[3] Crick F . Central dogma of molecular biology. Nature 1970;227:561–3. 4913914 10.1038/227561a0

[4] Robertson MP, Joyce GF. The origins of the RNA world. Cold Spring Harb Perspect Biol 2012;4:a003608. 20739415 10.1101/cshperspect.a003608 PMC3331698

[5] Orgel LE . Evolution of the genetic apparatus. J Mol Biol 1968;38:381–93. 5718557 10.1016/0022-2836(68)90393-8

[6] Woese CR, Dugre DH, Dugre SA, et al. On the fundamental nature and evolution of the genetic code. Cold Spring Harb Symp Quant Biol 1966;31:723–36. 5237212 10.1101/sqb.1966.031.01.093

[7] Woese CR, Dugre DH, Saxinger WC, Dugre SA. The molecular basis for the genetic code. Proc Natl Acad Sci U S A 1966;55:966–74. 5219702 10.1073/pnas.55.4.966 PMC224258

[8] Ban N, Nissen P, Hansen J, et al. The complete atomic structure of the large ribosomal subunit at 2.4 a resolution. Science 2000;289:905–20. 10937989 10.1126/science.289.5481.905

[9] Gilbert W . Origin of life: the RNA world. Nature 1986;319:618–8.

[10] Guerrier-Takada C, Gardiner K, Marsh T, et al. The RNA moiety of ribonuclease P is the catalytic subunit of the enzyme. Cell 1983;35:849–57. 6197186 10.1016/0092-8674(83)90117-4

[11] Kruger K, Grabowski PJ, Zaug AJ, et al. Self-splicing RNA: autoexcision and autocyclization of the ribosomal RNA intervening sequence of Tetrahymena. Cell 1982;31:147–57. 6297745 10.1016/0092-8674(82)90414-7

[12] Lewin R . RNA catalysis gives fresh perspective on the origin of life: the old chicken-and-egg problem of the origin of life is illuminated in unexpected ways by recent results on the splicing of RNA precursors. Science 1986;231:545–6. 17750962 10.1126/science.231.4738.545

[13] Pace NR, Marsh TL. RNA catalysis and the origin of life. Orig Life Evol Biosph 1985;16:97–116. 2423941 10.1007/BF01809465

[14] Shampo MA, Kyle RA, Steensma DP. Sidney Altman—Nobel laureate for work with RNA. Mayo Clin Proc 2012;87:e73. 23036683 10.1016/j.mayocp.2012.01.022 PMC3498233

[15] Sharp PA . On the origin of RNA splicing and introns. Cell 1985;42:397–400. 2411416 10.1016/0092-8674(85)90092-3

[16] Wimberly BT, Brodersen DE, Clemons WM, et al. Structure of the 30S ribosomal subunit. Nature 2000;407:327–39. 11014182 10.1038/35030006

[17] Yusupov MM, Yusupova GZ, Baucom A, et al. Crystal structure of the ribosome at 5.5 a resolution. Science 2001;292:883–96. 11283358 10.1126/science.1060089

[18] Palazzo AF, Lee ES. Non-coding RNA: what is functional and what is junk? Front Genet 2015;6:2. 25674102 10.3389/fgene.2015.00002 PMC4306305

[19] Pertea M . The human transcriptome: an unfinished story. Genes (Basel) 2012;3:344–60. 22916334 10.3390/genes3030344 PMC3422666

[20] Cabili MN, Trapnell C, Goff L, et al. Integrative annotation of human large intergenic noncoding RNAs reveals global properties and specific subclasses. Genes Dev 2011;25:1915–27. 21890647 10.1101/gad.17446611 PMC3185964

[21] Carninci P, Kasukawa T, Katayama S, et al. The transcriptional landscape of the mammalian genome. Science 2005;309:1559–63. 16141072 10.1126/science.1112014

[22] Guttman M, Amit I, Garber M, et al. Chromatin signature reveals over a thousand highly conserved large non-coding RNAs in mammals. Nature 2009;458:223–7. 19182780 10.1038/nature07672 PMC2754849

[23] Djebali S, Davis CA, Merkel A, et al. Landscape of transcription in human cells. Nature 2012;489:101–8. 22955620 10.1038/nature11233 PMC3684276

[24] Hangauer MJ, Vaughn IW, McManus MT. Pervasive transcription of the human genome produces thousands of previously unidentified long intergenic noncoding RNAs. PLoS Genet 2013;9:e1003569. 23818866 10.1371/journal.pgen.1003569 PMC3688513

[25] Wright PR, Mann M, Backofen R. Structure and interaction prediction in prokaryotic RNA biology. Microbiol Spectr 2018;6:6. 10.1128/microbiolspec.rwr-0001-2017 PMC11633574 29676245

[26] Slack FJ, Chinnaiyan AM. The role of non-coding RNAs in oncology. Cell 2019;179:1033–55. 31730848 10.1016/j.cell.2019.10.017 PMC7347159

[27] Frías-Lasserre D, Villagra CA. The importance of ncRNAs as epigenetic mechanisms in phenotypic variation and organic evolution. Front Microbiol 2017;8:2483. 10.3389/fmicb.2017.02483 PMC5744636 29312192

[28] Micheel J, Safrastyan A, Wollny D. Advances in non-coding RNA sequencing. Noncoding RNA 2021;7:70. 34842804 10.3390/ncrna7040070 PMC8628893

[29] Ikemura T, Dahlberg JE. Small ribonucleic acids of Escherichia coli. I. Characterization by polyacrylamide gel electrophoresis and fingerprint analysis. J Biol Chem 1973;248:5024–32. 4577761

[30] Lee RC, Feinbaum RL, Ambros V. The C. Elegans heterochronic gene lin-4 encodes small RNAs with antisense complementarity to lin-14. Cell 1993;75:843–54. 8252621 10.1016/0092-8674(93)90529-y

[31] Bhaskaran M, Mohan M. MicroRNAs: history, biogenesis, and their evolving role in animal development and disease. Vet Pathol 2014;51:759–74. 24045890 10.1177/0300985813502820 PMC4013251

[32] Kung JTY, Colognori D, Lee JT. Long noncoding RNAs: past, present, and future. Genetics 2013;193:651–69. 23463798 10.1534/genetics.112.146704 PMC3583990

[33] Zampetaki A, Albrecht A, Steinhofel K. Long non-coding RNA structure and function: is there a link? Front Physiol 2018;9:1201. 30197605 10.3389/fphys.2018.01201 PMC6117379

[34] Grote P, Wittler L, Währisch S, et al. The tissue-specific lncRNA Fendrr is an essential regulator of heart and body wall development in the mouse. Dev Cell 2013;24:206–14. 23369715 10.1016/j.devcel.2012.12.012 PMC4149175

[35] Guttman M, Donaghey J, Carey BW, et al. lincRNAs act in the circuitry controlling pluripotency and differentiation. Nature 2011;477:295–300. 21874018 10.1038/nature10398 PMC3175327

[36] Sauvageau M, Goff LA, Lodato S, et al. Multiple knockout mouse models reveal lincRNAs are required for life and brain development. Elife 2013;2:e01749. 10.7554/eLife.01749 PMC3874104 24381249

[37] Dawson WK, Bujnicki JM. Computational modeling of RNA 3D structures and interactions. Curr Opin Struct Biol 2016;37:22–8. 26689764 10.1016/j.sbi.2015.11.007

[38] Ambros V . The functions of animal microRNAs. Nature 2004;431:350–5. 15372042 10.1038/nature02871

[39] Fire A, Xu S, Montgomery MK, et al. Potent and specific genetic interference by double-stranded RNA in Caenorhabditis elegans. Nature 1998;391:806–11. 9486653 10.1038/35888

[40] Holley RW, Apgar J, Everett GA, et al. Structure of a ribonucleic acid. Science 1965;147:1462–5. 14263761 10.1126/science.147.3664.1462

[41] Holley RW, Everett GA, Madison JT, Zamir A. Nucleotide sequences in the yeast alanine transfer ribonucleic acid. J Biol Chem 1965;240:2122–8. 14299636

[42] Dieterich C, Stadler PF. Computational biology of RNA interactions. Wiley Interdiscip Rev RNA 2013;4:107–20. 23139167 10.1002/wrna.1147

[43] Helwak A, Kudla G, Dudnakova T, Tollervey D. Mapping the human miRNA interactome by CLASH reveals frequent noncanonical binding. Cell 2013;153:654–65. 23622248 10.1016/j.cell.2013.03.043 PMC3650559

[44] Graf J, Kretz M. From structure to function: route to understanding lncRNA mechanism. Bioessays 2020;42:e2000027. 33164244 10.1002/bies.202000027

[45] Kudla G, Granneman S, Hahn D, et al. Cross-linking, ligation, and sequencing of hybrids reveals RNA-RNA interactions in yeast. Proc Natl Acad Sci U S A 2011;108:10010–5. 21610164 10.1073/pnas.1017386108 PMC3116431

[46] Lu Z, Chang HY. The RNA Base-pairing problem and base-pairing solutions. Cold Spring Harb Perspect Biol 2018;10:a034926. 30510063 10.1101/cshperspect.a034926 PMC6280703

[47] Tsao N, Ashour ME, Mosammaparast N. How RNA impacts DNA repair. DNA Repair (Amst) 2023;131:103564. 37776841 10.1016/j.dnarep.2023.103564 PMC11232704

[48] Doudna JA, Charpentier E. Genome editing. The new frontier of genome engineering with CRISPR-Cas9. Science 2014;346:1258096. 25430774 10.1126/science.1258096

[49] Khelifi G, Hussein SMI. A new view of genome organization through RNA directed interactions. Frontiers in Cell and Developmental Biology 2020;8:8. 32760716 10.3389/fcell.2020.00517 PMC7371936

[50] Glisovic T, Bachorik JL, Yong J, Dreyfuss G. RNA-binding proteins and post-transcriptional gene regulation. FEBS Lett 2008;582:1977–86. 18342629 10.1016/j.febslet.2008.03.004 PMC2858862

[51] Will CL, Lührmann R. Spliceosome structure and function. Cold Spring Harb Perspect Biol 2011;3:a003707. 21441581 10.1101/cshperspect.a003707 PMC3119917

[52] Passmore LA, Coller J. Roles of mRNA poly(a) tails in regulation of eukaryotic gene expression. Nat Rev Mol Cell Biol 2022;23:93–106. 34594027 10.1038/s41580-021-00417-y PMC7614307

[53] Alberts B, Johnson A, Lewis J, et al. From RNA to protein. In: Molecular Biology of the Cell (4th ed). New York: Garland Science, 2002, pp. 132–3.

[54] Assmann SM, Chou H-L, Bevilacqua PC. Rock, scissors, paper: how RNA structure informs function. Plant Cell 2023;35:1671–707. 36747354 10.1093/plcell/koad026 PMC10226581

[55] Noller HF, Lancaster L, Zhou J, Mohan S. The ribosome moves: RNA mechanics and translocation. Nat Struct Mol Biol 2017;24:1021–7. 29215639 10.1038/nsmb.3505 PMC6581036

[56] Macfarlane L-A, Murphy PR. MicroRNA: biogenesis, function and role in cancer. Curr Genomics 2010;11:537–61. 21532838 10.2174/138920210793175895 PMC3048316

[57] Haruehanroengra P, Zheng YY, Zhou Y, et al. RNA modifications and cancer. RNA Biol 2020;17:1560–75. 31994439 10.1080/15476286.2020.1722449 PMC7567502

[58] Spencer M . The stereochemistry of deoxyribonucleic acid. II. Hydrogen-bonded pairs of bases. Acta Crystallogr 1959;12:66–71.

[59] Luttermann C, Meyers G. The importance of inter- and intramolecular base pairing for translation reinitiation on a eukaryotic bicistronic mRNA. Genes Dev 2009;23:331–44. 19204118 10.1101/gad.507609 PMC2648545

[60] Varani G, McClain, WH . The G·U wobble base pair. EMBO Rep 2000;1:18–23. 11256617 10.1093/embo-reports/kvd001 PMC1083677

[61] Brenner S . Codon-anticodon pairing: the wobble hypothesis. In: Molecular Biology: A Selection of Papers. Massachusetts: Academic Press, 2012, pp. 370–7.

[62] Murphy FV, Ramakrishnan V. Structure of a purine-purine wobble base pair in the decoding center of the ribosome. Nat Struct Mol Biol 2004;11:1251–2. 15558050 10.1038/nsmb866

[63] Appasamy SD, Hamdani HY, Ramlan EI, Firdaus-Raih M. InterRNA: a database of base interactions in RNA structures. Nucleic Acids Res 2016;44:D266–71. 26553798 10.1093/nar/gkv1186 PMC4702846

[64] Treeck BV, Protter DSW, Matheny T, et al. RNA self-assembly contributes to stress granule formation and defining the stress granule transcriptome. PNAS 2018;115:2734–9. 29483269 10.1073/pnas.1800038115 PMC5856561

[65] Van Treeck B, Parker R. Emerging roles for intermolecular RNA-RNA interactions in RNP assemblies. Cell 2018;174:791–802. 30096311 10.1016/j.cell.2018.07.023 PMC6200146

[66] Lim LP, Lau NC, Garrett-Engele P, et al. Microarray analysis shows that some microRNAs downregulate large numbers of target mRNAs. Nature 2005;433:769–73. 15685193 10.1038/nature03315

[67] Friedman RC, Farh KK-H, Burge CB, Bartel DP. Most mammalian mRNAs are conserved targets of microRNAs. Genome Res 2009;19:92–105. 18955434 10.1101/gr.082701.108 PMC2612969

[68] Neph S, Vierstra J, Stergachis AB, et al. An expansive human regulatory lexicon encoded in transcription factor footprints. Nature 2012;489:83–90. 22955618 10.1038/nature11212 PMC3736582

[69] Buvoli M, Cobianchi F, Riva S. Interaction of hnRNP A1 with snRNPs and pre-mRNAs: evidence for a possible role of A1 RNA annealing activity in the first steps of spliceosome assembly. Nucleic Acids Res 1992;20:5017–25. 1329035 10.1093/nar/20.19.5017 PMC334278

[70] Wahl MC, Will CL, Lührmann R. The spliceosome: design principles of a dynamic RNP machine. Cell 2009;136:701–18. 19239890 10.1016/j.cell.2009.02.009

[71] Seraphin B, Rosbash M. Identification of functional U1 snRNA-pre-mRNA complexes committed to spliceosome assembly and splicing. Cell 1989;59:349–58. 2529976 10.1016/0092-8674(89)90296-1

[72] Matera AG, Terns RM, Terns MP. Non-coding RNAs: lessons from the small nuclear and small nucleolar RNAs. Nat Rev Mol Cell Biol 2007;8:209–20. 17318225 10.1038/nrm2124

[73] Bachellerie JP, Cavaillé J, Hüttenhofer A. The expanding snoRNA world. Biochimie 2002;84:775–90. 12457565 10.1016/s0300-9084(02)01402-5

[74] Kable ML, Seiwert SD, Heidmann S, Stuart K. RNA editing: a mechanism for gRNA-specified uridylate insertion into precursor mRNA. Science 1996;273:1189–95. 8703045 10.1126/science.273.5279.1189

[75] Evans D, Marquez SM, Pace NR. RNase P: interface of the RNA and protein worlds. Trends Biochem Sci 2006;31:333–41. 16679018 10.1016/j.tibs.2006.04.007

[76] Alberts B, Johnson A, Lewis J, et al. The initiation and completion of DNA replication in chromosomes. In: Molecular Biology of the Cell (4th edition). New York: Garland Science, 2002, pp. 660–2.

[77] Schneider B, Morávek Z, Berman HM. RNA conformational classes. Nucleic Acids Res 2004;32:1666–77. 15016910 10.1093/nar/gkh333 PMC390331

[78] Schroeder SJ . Challenges and approaches to predicting RNA with multiple functional structures. RNA 2018;24:1615–24. 30143552 10.1261/rna.067827.118 PMC6239171

[79] Shajani Z, Varani G. NMR studies of dynamics in RNA and DNA by 13C relaxation. Biopolymers 2007;86:348–59. 17154290 10.1002/bip.20650

[80] Mustoe AM, Brooks CL, Al-Hashimi HM. Hierarchy of RNA functional dynamics. Annu Rev Biochem 2014;83:441–66. 24606137 10.1146/annurev-biochem-060713-035524 PMC4048628

[81] Al-Hashimi HM . Beyond static structures of RNA by NMR: folding, refolding, and dynamics at atomic resolution. Biopolymers 2007;86:345–7. 17597469 10.1002/bip.20754

[82] Vicens Q, Kieft JS. Thoughts on how to think (and talk) about RNA structure. Proc Natl Acad Sci 2022;119:e2112677119. 35439059 10.1073/pnas.2112677119 PMC9169933

[83] Zhang J, Fei Y, Sun L, Zhang QC. Advances and opportunities in RNA structure experimental determination and computational modeling. Nat Methods 2022;19:1193–207. 36203019 10.1038/s41592-022-01623-y

[84] Dai X, Zhang S, Zaleta-Rivera K. RNA: interactions drive functionalities. Mol Biol Rep 2020;47:1413–34. 31838657 10.1007/s11033-019-05230-7 PMC7089156

[85] Meyer IM . Predicting novel RNA–RNA interactions. Curr Opin Struct Biol 2008;18:387–93. 18485695 10.1016/j.sbi.2008.03.006

[86] Umu SU, Gardner PP. A comprehensive benchmark of RNA–RNA interaction prediction tools for all domains of life. Bioinformatics 2017;33:988–96. 27993777 10.1093/bioinformatics/btw728 PMC5408919

[87] Lai D, Meyer IM. A comprehensive comparison of general RNA–RNA interaction prediction methods. Nucleic Acids Res 2016;44:e61. 26673718 10.1093/nar/gkv1477 PMC4838349

[88] Matarrese MAG, Loppini A,Nicoletti M, et al. Assessment of tools for RNA secondary structure prediction and extraction: a final-user perspective. J Biomol Struct Dyn 2023;41:6917–36. 10.1080/07391102.2022.2116110 36106933

[89] Barnwal RP, Yang F, Varani G. Applications of NMR to structure determination of RNAs large and small. Arch Biochem Biophys 2017;628:42–56. 28600200 10.1016/j.abb.2017.06.003 PMC5555312

[90] Li B, Cao Y, Westhof E, Miao Z. Advances in RNA 3D structure Modeling using experimental data. Front Genet 2020;11:574485. 10.3389/fgene.2020.574485 PMC7649352 33193680

[91] Krishnan V, Rupp B. Macromolecular structure determination: comparison of X-ray crystallography and NMR spectroscopy. In: Encyclopedia of Life Sciences. New Jersey: John Wiley & Sons, 2012, a0002716.

[92] Fürtig B, Richter C, Wöhnert J, Schwalbe H. NMR spectroscopy of RNA. Chembiochem 2003;4:936–62. 14523911 10.1002/cbic.200300700

[93] Westhof E . Twenty years of RNA crystallography. RNA 2015;21:486–7. 25780106 10.1261/rna.049726.115 PMC4371248

[94] Consortium TR . RNAcentral: a comprehensive database of non-coding RNA sequences. Nucleic Acids Res 2017;45:D128–34. 27794554 10.1093/nar/gkw1008 PMC5210518

[95] Kretz M, Siprashvili Z, Chu C, et al. Control of somatic tissue differentiation by the long non-coding RNA TINCR. Nature 2013;493:231–5. 23201690 10.1038/nature11661 PMC3674581

[96] Engreitz JM, Sirokman K, McDonel P, et al. RNA-RNA interactions enable specific targeting of noncoding RNAs to nascent pre-mRNAs and chromatin sites. Cell 2014;159:188–99. 25259926 10.1016/j.cell.2014.08.018 PMC4177037

[97] Nguyen TC, Cao X, Yu P, et al. Mapping RNA–RNA interactome and RNA structure in vivo by MARIO. Nat Commun 2016;7:1–12. 10.1038/ncomms12023 PMC4931010 27338251

[98] Lu Z, Zhang QC, Lee B, et al. RNA duplex map in living cells reveals higher-order transcriptome structure. Cell 2016;165:1267–79. 27180905 10.1016/j.cell.2016.04.028 PMC5029792

[99] Aw JGA, Shen Y, Wilm A, et al. In vivo mapping of eukaryotic RNA Interactomes reveals principles of higher-order organization and regulation. Mol Cell 2016;62:603–17. 27184079 10.1016/j.molcel.2016.04.028

[100] Sharma E, Sterne-Weiler T, O’Hanlon D, Blencowe BJ. Global mapping of human RNA-RNA interactions. Mol Cell 2016;62:618–26. 27184080 10.1016/j.molcel.2016.04.030

[101] Yi Y, Zhao Y, Li C, et al. RAID v2.0: an updated resource of RNA-associated interactions across organisms. Nucleic Acids Res 2017;45:D115–8. 27899615 10.1093/nar/gkw1052 PMC5210540

[102] Yuan J, Wu W, Xie C, et al. NPInter v2.0: an updated database of ncRNA interactions. Nucleic Acids Res 2014;42:D104–8. 24217916 10.1093/nar/gkt1057 PMC3965026

[103] Teng X, Chen X, Xue H, et al. NPInter v4.0: an integrated database of ncRNA interactions. Nucleic Acids Res 2020;48:D160–5. 31670377 10.1093/nar/gkz969 PMC7145607

[104] Wu T, Wang J, Liu C, et al. NPInter: the noncoding RNAs and protein related biomacromolecules interaction database. Nucleic Acids Res 2006;34:D150–2. 16381834 10.1093/nar/gkj025 PMC1347388

[105] Kang J, Tang Q, He J, et al. RNAInter v4.0: RNA interactome repository with redefined confidence scoring system and improved accessibility. Nucleic Acids Res 2021;50:D326–32. 10.1093/nar/gkab997 PMC8728132 34718726

[106] Lin Y, Liu T, Cui T, et al. RNAInter in 2020: RNA interactome repository with increased coverage and annotation. Nucleic Acids Res 2020;48:D189–97. 31906603 10.1093/nar/gkz804 PMC6943043

[107] Gong J, Shao D, Xu K, et al. RISE: a database of RNA interactome from sequencing experiments. Nucleic Acids Res 2018;46:D194–201. 29040625 10.1093/nar/gkx864 PMC5753368

[108] Iyer MK, Niknafs YS, Malik R, et al. The landscape of long noncoding RNAs in the human transcriptome. Nat Genet 2015;47:199–208. 25599403 10.1038/ng.3192 PMC4417758

[109] Trotta E . On the normalization of the minimum free energy of RNAs by sequence length. PloS One 2014;9:e113380. 25405875 10.1371/journal.pone.0113380 PMC4236180

[110] Sykes MT, Levitt M. Simulations of RNA base pairs in a nanodroplet reveal solvation-dependent stability. Proc Natl Acad Sci 2007;104:12336–40. 17636124 10.1073/pnas.0705573104 PMC1920539

[111] Parsch J, Braverman JM, Stephan W. Comparative sequence analysis and patterns of covariation in RNA secondary structures. Genetics 2000;154:909–21. 10655240 10.1093/genetics/154.2.909 PMC1460946

[112] Sato K, Akiyama M, Sakakibara Y. RNA secondary structure prediction using deep learning with thermodynamic integration. Nat Commun 2021;12:941. 33574226 10.1038/s41467-021-21194-4 PMC7878809

[113] Zhang H, Zhang C, Li Z, et al. A new method of RNA secondary structure prediction based on convolutional neural network and dynamic programming. Front Genet 2019;10:467. 31191603 10.3389/fgene.2019.00467 PMC6540740

[114] Mathews DH, Turner DH. Prediction of RNA secondary structure by free energy minimization. Curr Opin Struct Biol 2006;16:270–8. 16713706 10.1016/j.sbi.2006.05.010

[115] Tinoco I, Borer PN, Dengler B, et al. Improved estimation of secondary structure in ribonucleic acids. Nat New Biol 1973;246:40–1. 4519026 10.1038/newbio246040a0

[116] Borer PN, Dengler B, Tinoco I, Uhlenbeck OC. Stability of ribonucleic acid double-stranded helices. J Mol Biol 1974;86:843–53. 4427357 10.1016/0022-2836(74)90357-x

[117] Turner DH, Mathews DH. NNDB: the nearest neighbor parameter database for predicting stability of nucleic acid secondary structure. Nucleic Acids Res 2010;38:D280–2. 19880381 10.1093/nar/gkp892 PMC2808915

[118] Mathews DH, Sabina J, Zuker M, Turner DH. Expanded sequence dependence of thermodynamic parameters improves prediction of RNA secondary structure. J Mol Biol 1999;288:911–40. 10329189 10.1006/jmbi.1999.2700

[119] Bellman R . The theory of dynamic programming. Bull Amer Math Soc 1954;60:503–15.

[120] Bellman R . The structure of dynamic programming processes. In: Dynamic Programming (6th Ed). New Jersey: Princeton University Press, 1957, pp. 81–115.

[121] Nussinov R, Pieczenik G, Griggs JR, Kleitman DJ. Algorithms for loop matchings. SIAM J Appl Math 1978;35:68–82.

[122] Lyngsø RB . RNA secondary structure prediction by minimum free energy. In: Encyclopedia of Algorithms. Germany: SpringerLink, 2016, pp. 1846–50.

[123] Zuker M, Stiegler P. Optimal computer folding of large RNA sequences using thermodynamics and auxiliary information. Nucleic Acids Res 1981;9:133–48. 6163133 10.1093/nar/9.1.133 PMC326673

[124] Backofen R, Hess WR. Computational prediction of sRNAs and their targets in bacteria. RNA Biol 2010;7:33–42. 20061798 10.4161/rna.7.1.10655

[125] Lorenz R, Bernhart SH, Höner zu Siederdissen C, et al. ViennaRNA package 2.0. Algorithms for Molecular Biology 2011;6:26. 22115189 10.1186/1748-7188-6-26 PMC3319429

[126] Rehmsmeier M, Steffen P, Hochsmann M, et al. Fast and effective prediction of microRNA/target duplexes. RNA 2004;10:1507–17. 15383676 10.1261/rna.5248604 PMC1370637

[127] Tjaden B . TargetRNA: a tool for predicting targets of small RNA action in bacteria. Nucleic Acids Res 2008;36:W109–13. 18477632 10.1093/nar/gkn264 PMC2447797

[128] Tafer H, Hofacker IL. RNAplex: a fast tool for RNA–RNA interaction search. Bioinformatics 2008;24:2657–63. 18434344 10.1093/bioinformatics/btn193

[129] Wenzel A, Akbaşli E, Gorodkin J. RIsearch: fast RNA–RNA interaction search using a simplified nearest-neighbor energy model. Bioinformatics 2012;28:2738–46. 22923300 10.1093/bioinformatics/bts519 PMC3476332

[130] Gerlach W, Giegerich R. GUUGle: a utility for fast exact matching under RNA complementary rules including G–U base pairing. Bioinformatics 2006;22:762–4. 16403789 10.1093/bioinformatics/btk041

[131] McCaskill JS . The equilibrium partition function and base pair binding probabilities for RNA secondary structure. Biopolymers 1990;29:1105–19. 1695107 10.1002/bip.360290621

[132] Ding Y, Chan CY, Lawrence CE. Sfold web server for statistical folding and rational design of nucleic acids. Nucleic Acids Res 2004;32:W135–41. 15215366 10.1093/nar/gkh449 PMC441587

[133] Mückstein U, Tafer H, Hackermüller J, et al. Thermodynamics of RNA-RNA binding. Bioinformatics 2006;22:1177–82. 16446276 10.1093/bioinformatics/btl024

[134] Mann M, Wright PR, Backofen R. IntaRNA 2.0: enhanced and customizable prediction of RNA–RNA interactions. Nucleic Acids Res 2017;45:W435–9. 28472523 10.1093/nar/gkx279 PMC5570192

[135] Busch A, Richter AS, Backofen R. IntaRNA: efficient prediction of bacterial sRNA targets incorporating target site accessibility and seed regions. Bioinformatics 2008;24:2849–56. 18940824 10.1093/bioinformatics/btn544 PMC2639303

[136] Eggenhofer F, Tafer H, Stadler PF, Hofacker IL. RNApredator: fast accessibility-based prediction of sRNA targets. Nucleic Acids Res 2011;39:W149–54. 21672960 10.1093/nar/gkr467 PMC3125805

[137] Lu ZJ, Mathews DH. OligoWalk: an online siRNA design tool utilizing hybridization thermodynamics. Nucleic Acids Res 2008;36:W104–8. 18490376 10.1093/nar/gkn250 PMC2447759

[138] Poolsap U, Kato Y, Sato K, et al. Using binding profiles to predict binding sites of target RNAs. J Bioinform Comput Biol 2011;09:697–713. 10.1142/s0219720011005628 22084009

[139] Salari R, Backofen R, Sahinalp SC. Fast prediction of RNA-RNA interaction. Algorithms for Molecular Biology 2010;5:5. 20047661 10.1186/1748-7188-5-5 PMC2828455

[140] Alkan F, Wenzel A, Palasca O, et al. RIsearch2: suffix array-based large-scale prediction of RNA-RNA interactions and siRNA off-targets. Nucleic Acids Res 2017;45:e60. 28108657 10.1093/nar/gkw1325 PMC5416843

[141] Fukunaga T, Hamada M. RIblast: an ultrafast RNA-RNA interaction prediction system based on a seed-and-extension approach. Bioinformatics 2017;33:2666–74. 28459942 10.1093/bioinformatics/btx287 PMC5860064

[142] Kery MB, Feldman M, Livny J, Tjaden B. TargetRNA2: identifying targets of small regulatory RNAs in bacteria. Nucleic Acids Res 2014;42:W124–9. 24753424 10.1093/nar/gku317 PMC4086111

[143] Andronescu M, Aguirre-Hernández R, Condon A, Hoos HH. RNAsoft: a suite of RNA secondary structure prediction and design software tools. Nucleic Acids Res 2003;31:3416–22. 12824338 10.1093/nar/gkg612 PMC169018

[144] Andronescu M, Zhang ZC, Condon A. Secondary structure prediction of interacting RNA molecules. J Mol Biol 2005;345:987–1001. 15644199 10.1016/j.jmb.2004.10.082

[145] Lorenz R, Hofacker IL, Stadler PF. RNA folding with hard and soft constraints. Algorithms Mol Biol 2016;11:8. 27110276 10.1186/s13015-016-0070-z PMC4842303

[146] Markham NR, Zuker M. UNAFold: software for nucleic acid folding and hybridization. Methods Mol Biol 2008;453:3–31. 18712296 10.1007/978-1-60327-429-6_1

[147] Schäfer RA, Voß B. RNAnue: efficient data analysis for RNA–RNA interactomics. Nucleic Acids Res 2021;49:5493–501. 34019662 10.1093/nar/gkab340 PMC8191800

[148] Fornace ME, Porubsky NJ, Pierce NA. A unified dynamic programming framework for the analysis of interacting nucleic acid strands: enhanced models, scalability, and speed. ACS Synthetic Biology 2020;9:2665–78. 32910644 10.1021/acssynbio.9b00523

[149] Zadeh JN, Steenberg CD, Bois JS, et al. NUPACK: analysis and design of nucleic acid systems. J Comput Chem 2011;32:170–3. 20645303 10.1002/jcc.21596

[150] Hofacker IL, Stadler PF. Memory efficient folding algorithms for circular RNA secondary structures. Bioinformatics 2006;22:1172–6. 16452114 10.1093/bioinformatics/btl023

[151] Hofacker IL, Fontana W, Stadler PF, et al. Fast folding and comparison of RNA secondary structures. Monatsh Chem 1994;125:167–88.

[152] Zuker M . Mfold web server for nucleic acid folding and hybridization prediction. Nucleic Acids Res 2003;31:3406–15. 12824337 10.1093/nar/gkg595 PMC169194

[153] Alkan C, Karakoç E, Nadeau JH, et al. RNA-RNA interaction prediction and antisense RNA target search. J Comput Biol 2006;13:267–82. 16597239 10.1089/cmb.2006.13.267

[154] Chitsaz H, Salari R, Sahinalp SC, Backofen R. A partition function algorithm for interacting nucleic acid strands. Bioinformatics 2009;25:i365–73. 19478011 10.1093/bioinformatics/btp212 PMC2687966

[155] Salari R, Backofen R, Sahinalp SC. Fast prediction of RNA-RNA interaction. Algorithms Mol Biol 2010;5:5. 10.1186/1748-7188-5-5 PMC2828455 20047661

[156] Pervouchine DD . IRIS: intermolecular RNA interaction search. Genome Inform 2004;15:92–101. 15706495

[157] Huang FWD, Qin J, Reidys CM, Stadler PF. Target prediction and a statistical sampling algorithm for RNA–RNA interaction. Bioinformatics 2010;26:175–81. 19910305 10.1093/bioinformatics/btp635 PMC2804298

[158] Huang FWD, Qin J, Reidys CM, Stadler PF. Partition function and base pairing probabilities for RNA-RNA interaction prediction. Bioinformatics 2009;25:2646–54. 19671692 10.1093/bioinformatics/btp481

[159] Bernhart SH, Tafer H, Mückstein U, et al. Partition function and base pairing probabilities of RNA heterodimers. Algorithms Mol Biol 2006;1:3. 16722605 10.1186/1748-7188-1-3 PMC1459172

[160] Dirks RM, Bois JS, Schaeffer JM, et al. Thermodynamic analysis of interacting nucleic acid strands. SIAM Review 2007;49:65–88.

[161] Ebrahimpour-Boroojeny A, Rajopadhye S, Chitsaz H. BPPart: RNA-RNA Interaction Partition Function in the Absence of Entropy. In: 21st International Workshop on Algorithms in Bioinformatics, virtual. Germany: Leibniz International Proceedings in Informatics, 2021, 201:14:1-14:24.

[162] Kato Y, Akutsu T, Seki H. A grammatical approach to RNA–RNA interaction prediction. Pattern Recognition 2009;42:531–8.

[163] Kato Y, Sato K, Hamada M, et al. RactIP: fast and accurate prediction of RNA-RNA interaction using integer programming. Bioinformatics 2010;26:i460–6. 20823308 10.1093/bioinformatics/btq372 PMC2935440

[164] Gardner PP, Giegerich R. A comprehensive comparison of comparative RNA structure prediction approaches. BMC Bioinformatics 2004;5:140. 15458580 10.1186/1471-2105-5-140 PMC526219

[165] Appasamy SD, Ramlan EI, Firdaus-Raih M. Comparative sequence and structure analysis reveals the conservation and diversity of nucleotide positions and their associated tertiary interactions in the riboswitches. PloS One 2013;8:e73984. 24040136 10.1371/journal.pone.0073984 PMC3764141

[166] Madison JT, Everett GA, Kung H. Nucleotide sequence of a yeast tyrosine transfer RNA. Science 1966;153:531–4. 5938777 10.1126/science.153.3735.531

[167] Gutell RR, Lee JC, Cannone JJ. The accuracy of ribosomal RNA comparative structure models. Curr Opin Struct Biol 2002;12:301–10. 12127448 10.1016/s0959-440x(02)00339-1

[168] Knudsen B, Hein J. RNA secondary structure prediction using stochastic context-free grammars and evolutionary history. Bioinformatics 1999;15:446–54. 10383470 10.1093/bioinformatics/15.6.446

[169] Larkin MA, Blackshields G, Brown NP, et al. Clustal W and Clustal X version 2.0. Bioinformatics 2007;23:2947–8. 17846036 10.1093/bioinformatics/btm404

[170] Thompson JD, Gibson TJ, Higgins DG. Multiple sequence alignment using ClustalW and ClustalX. Curr Protoc Bioinformatics 2002;Chapter 2:Unit 2.3. 10.1002/0471250953.bi0203s00 18792934

[171] Katoh K, Standley DM. MAFFT multiple sequence alignment software version 7: improvements in performance and usability. Mol Biol Evol 2013;30:772–80. 23329690 10.1093/molbev/mst010 PMC3603318

[172] Bernhart SH, Hofacker IL, Will S, et al. RNAalifold: improved consensus structure prediction for RNA alignments. BMC Bioinformatics 2008;9:474. 19014431 10.1186/1471-2105-9-474 PMC2621365

[173] Knudsen B, Hein J. Pfold: RNA secondary structure prediction using stochastic context-free grammars. Nucleic Acids Res 2003;31:3423–8. 12824339 10.1093/nar/gkg614 PMC169020

[174] Seemann SE, Menzel P, Backofen R, Gorodkin J. The PETfold and PETcofold web servers for intra- and intermolecular structures of multiple RNA sequences. Nucleic Acids Res 2011;39:W107–11. 21609960 10.1093/nar/gkr248 PMC3125731

[175] Sankoff D . Simultaneous solution of the RNA folding, alignment and protosequence problems. SIAM J Appl Math 1985;45:810–25.

[176] Tahi F, Gouy M, Régnier M. Automatic RNA secondary structure prediction with a comparative approach. Comput Chem 2002;26:521–30. 12144180 10.1016/s0097-8485(02)00012-8

[177] Tahi F, Engelen S, Regnier M. A fast algorithm for RNA secondary structure prediction including pseudoknots. Third IEEE Symposium on Bioinformatics and Bioengineering, Bethesda, MD, 2003. Proceedings. 2003; 11–7. IEEE Computer Society, Washington, DC, USA.

[178] Havgaard JH, Gorodkin J. RNA structural alignments, part I: Sankoff-based approaches for structural alignments. Methods Mol Biol 2014;1097:275–90. 24639164 10.1007/978-1-62703-709-9_13

[179] Höchsmann M, Töller T, Giegerich R, Kurtz S. Local similarity in RNA secondary structures. Proc IEEE Comput Soc Bioinform Conf 2003;2:159–68. 16452790

[180] Gorodkin J, Heyer LJ, Stormo GD. Finding the most significant common sequence and structure motifs in a set of RNA sequences. Nucleic Acids Res 1997;25:3724–32. 9278497 10.1093/nar/25.18.3724 PMC146942

[181] Havgaard JH, Lyngsø RB, Stormo GD, et al. Pairwise local structural alignment of RNA sequences with sequence similarity less than 40%. Bioinformatics 2005;21:1815–24. 15657094 10.1093/bioinformatics/bti279

[182] Sundfeld D, Havgaard JH, de Melo ACMA, Gorodkin J. Foldalign 2.5: multithreaded implementation for pairwise structural RNA alignment. Bioinformatics 2016;32:1238–40. 26704597 10.1093/bioinformatics/btv748 PMC4824132

[183] Mathews DH, Turner DH. Dynalign: an algorithm for finding the secondary structure common to two RNA sequences. J Mol Biol 2002;317:191–203. 11902836 10.1006/jmbi.2001.5351

[184] Bradley RK, Pachter L, Holmes I. Specific alignment of structured RNA: stochastic grammars and sequence annealing. Bioinformatics 2008;24:2677–83. 18796475 10.1093/bioinformatics/btn495 PMC2732270

[185] Tabei Y, Kiryu H, Kin T, Asai K. A fast structural multiple alignment method for long RNA sequences. BMC Bioinformatics 2008;9:33. 18215258 10.1186/1471-2105-9-33 PMC2375124

[186] Hofacker IL, Bernhart SHF, Stadler PF. Alignment of RNA base pairing probability matrices. Bioinformatics 2004;20:2222–7. 15073017 10.1093/bioinformatics/bth229

[187] Will S, Reiche K, Hofacker IL, et al. Inferring noncoding RNA families and classes by means of genome-scale structure-based clustering. PLoS Comput Biol 2007;3:e65. 17432929 10.1371/journal.pcbi.0030065 PMC1851984

[188] Torarinsson E, Havgaard JH, Gorodkin J. Multiple structural alignment and clustering of RNA sequences. Bioinformatics 2007;23:926–32. 17324941 10.1093/bioinformatics/btm049

[189] Kiryu H, Tabei Y, Kin T, Asai K. Murlet: a practical multiple alignment tool for structural RNA sequences. Bioinformatics 2007;23:1588–98. 17459961 10.1093/bioinformatics/btm146

[190] Dalli D, Wilm A, Mainz I, Steger G. StrAl: progressive alignment of non-coding RNA using base pairing probability vectors in quadratic time. Bioinformatics 2006;22:1593–9. 16613908 10.1093/bioinformatics/btl142

[191] Do CB, Foo C-S, Batzoglou S. A max-margin model for efficient simultaneous alignment and folding of RNA sequences. Bioinformatics 2008;24:i68–76. 18586747 10.1093/bioinformatics/btn177 PMC2718655

[192] Allali J, Sagot M-F. A new distance for high level RNA secondary structure comparison. IEEE/ACM Trans Comput Biol Bioinform 2005;2:3–14. 17044160 10.1109/TCBB.2005.2

[193] Reeder J, Giegerich R. Consensus shapes: an alternative to the Sankoff algorithm for RNA consensus structure prediction. Bioinformatics 2005;21:3516–23. 16020472 10.1093/bioinformatics/bti577

[194] Bossanyi M-A, Carpentier V, Glouzon J-PS, et al. aliFreeFoldMulti: alignment-free method to predict secondary structures of multiple RNA homologs. NAR Genomics and Bioinformatics 2020;2:lqaa086. 33575631 10.1093/nargab/lqaa086 PMC7671329

[195] Notredame C, Higgins DG, Heringa J. T-coffee: a novel method for fast and accurate multiple sequence alignment. J Mol Biol 2000;302:205–17. 10964570 10.1006/jmbi.2000.4042

[196] Bremges A, Schirmer S, Giegerich R. Fine-tuning structural RNA alignments in the twilight zone. BMC Bioinformatics 2010;11:222. 20433706 10.1186/1471-2105-11-222 PMC2876130

[197] Lindgreen S, Gardner PP, Krogh A. MASTR: multiple alignment and structure prediction of non-coding RNAs using simulated annealing. Bioinformatics 2007;23:3304–11. 18006551 10.1093/bioinformatics/btm525

[198] Xu X, Ji Y, Stormo GD. RNA sampler: a new sampling based algorithm for common RNA secondary structure prediction and structural alignment. Bioinformatics 2007;23:1883–91. 17537756 10.1093/bioinformatics/btm272

[199] Yao Z, Weinberg Z, Ruzzo WL. CMfinder—a covariance model based RNA motif finding algorithm. Bioinformatics 2006;22:445–52. 16357030 10.1093/bioinformatics/btk008

[200] Bauer M, Klau GW, Reinert K. Accurate multiple sequence-structure alignment of RNA sequences using combinatorial optimization. BMC Bioinformatics 2007;8:271. 17662141 10.1186/1471-2105-8-271 PMC1955456

[201] Li AX, Marz M, Qin J, Reidys CM. RNA-RNA interaction prediction based on multiple sequence alignments. Bioinformatics 2011;27:456–63. 21134894 10.1093/bioinformatics/btq659

[202] Richter AS, Backofen R. Accessibility and conservation. RNA Biol 2012;9:954–65. 22767260 10.4161/rna.20294 PMC3495738

[203] Krüger J, Rehmsmeier M. RNAhybrid: microRNA target prediction easy, fast and flexible. Nucleic Acids Res 2006;34:W451–4. 16845047 10.1093/nar/gkl243 PMC1538877

[204] Hartung J . A note on combining dependent tests of significance. Biom J 1999;41:849–55.

[205] Wright PR, Georg J, Mann M, et al. CopraRNA and IntaRNA: predicting small RNA targets, networks and interaction domains. Nucleic Acids Res 2014;42:W119–23. 24838564 10.1093/nar/gku359 PMC4086077

[206] Brierley I, Pennell S, Gilbert RJC. Viral RNA pseudoknots: versatile motifs in gene expression and replication. Nat Rev Microbiol 2007;5:598–610. 17632571 10.1038/nrmicro1704 PMC7096944

[207] Puglisi JD, Wyatt JR, Tinoco I. Conformation of an RNA pseudoknot. J Mol Biol 1990;214:437–53. 1696318 10.1016/0022-2836(90)90192-O PMC7131512

[208] Chiaruttini C, Milet M, Springer M. A long-range RNA-RNA interaction forms a pseudoknot required for translational control of the IF3-L35-L20 ribosomal protein operon in Escherichia coli. EMBO J 1996;15:4402–13. 8861967 PMC452164

[209] Ly H, Xu L, Rivera MA, et al. A role for a novel ‘trans-pseudoknot’ RNA–RNA interaction in the functional dimerization of human telomerase. Genes Dev 2003;17:1078–83. 12730131 10.1101/gad.1060803 PMC196051

[210] Staple DW, Butcher SE. Pseudoknots: RNA structures with diverse functions. PLoS Biol 2005;3:e213. 15941360 10.1371/journal.pbio.0030213 PMC1149493

[211] Ten Dam E, Pleij K, Draper D. Structural and functional aspects of RNA pseudoknots. Biochemistry 1992;31:11665–76. 1280160 10.1021/bi00162a001

[212] Pleij CWA . Pseudoknots: a new motif in the RNA game. Trends Biochem Sci 1990;15:143–7. 1692647 10.1016/0968-0004(90)90214-v

[213] Pleij CWA, Bosch L. [21] RNA pseudoknot: structure, detection, and prediction. Methods Enzymol 1989;180:289–303. 2482419 10.1016/0076-6879(89)80107-7

[214] Nebel ME, Weinberg F. Algebraic and combinatorial properties of common RNA pseudoknot classes with applications. J Comput Biol 2012;19:1134–50. 23057823 10.1089/cmb.2011.0094 PMC3469209

[215] Rivas E, Eddy SR. A dynamic programming algorithm for RNA structure prediction including pseudoknots. J Mol Biol 1999;285:2053–68. 9925784 10.1006/jmbi.1998.2436

[216] Ren J, Rastegari B, Condon A, et al. HotKnots: heuristic prediction of RNA secondary structures including pseudoknots. RNA 2005;11:1494–504. 16199760 10.1261/rna.7284905 PMC1370833

[217] Cary RB, Stormo GD. Graph-theoretic approach to RNA modeling using comparative data. Proc Int Conf Intell Syst Mol Biol 1995;3:75–80. 7584469

[218] Page RDM . Comparative analysis of secondary structure of insect mitochondrial small subunit ribosomal RNA using maximum weighted matching. Nucleic Acids Res 2000;28:3839–45. 11024161 10.1093/nar/28.20.3839 PMC110784

[219] Tabaska JE, Cary RB, Gabow HN, Stormo GD. An RNA folding method capable of identifying pseudoknots and base triples. Bioinformatics 1998;14:691–9. 9789095 10.1093/bioinformatics/14.8.691

[220] Ruan J, Stormo GD, Zhang W. An iterated loop matching approach to the prediction of RNA secondary structures with pseudoknots. Bioinformatics 2003;20:58–66. 10.1093/bioinformatics/btg373 14693809

[221] Ruan J, Stormo GD, Zhang W. ILM: a web server for predicting RNA secondary structures with pseudoknots. Nucleic Acids Res 2004;32:W146–9. 15215368 10.1093/nar/gkh444 PMC441582

[222] Chen X, He S-M, Bu D, et al. FlexStem: improving predictions of RNA secondary structures with pseudoknots by reducing the search space. Bioinformatics 2008;24:1994–2001. 18586700 10.1093/bioinformatics/btn327

[223] Xayaphoummine A, Bucher T, Isambert H. Kinefold web server for RNA/DNA folding path and structure prediction including pseudoknots and knots. Nucleic Acids Res 2005;33:W605–10. 15980546 10.1093/nar/gki447 PMC1160208

[224] Bindewald E, Afonin K, Jaeger L, Shapiro BA. Multi-strand RNA secondary structure prediction and nanostructure design including pseudoknots. ACS Nano 2011;5:9542–51. 22067111 10.1021/nn202666w PMC3263976

[225] Xu X, Chen S-J. VfoldCPX server: predicting RNA-RNA complex structure and stability. PloS One 2016;11:e0163454. 27657918 10.1371/journal.pone.0163454 PMC5033388

[226] Sato K, Kato Y, Hamada M, et al. IPknot: fast and accurate prediction of RNA secondary structures with pseudoknots using integer programming. Bioinformatics 2011;27:i85–93. 21685106 10.1093/bioinformatics/btr215 PMC3117384

[227] Gultyaev AP, van Batenburg FH, Pleij CW. The computer simulation of RNA folding pathways using a genetic algorithm. J Mol Biol 1995;250:37–51. 7541471 10.1006/jmbi.1995.0356

[228] Shi Y-Z, Wu Y-Y, Feng-Hua W, et al. RNA structure prediction: progress and perspective. Chinese Physics B 2014;23:078701.

[229] Turner DH . Thermodynamics of base pairing. Curr Opin Struct Biol 1996;6:299–304. 8804832 10.1016/s0959-440x(96)80047-9

[230] Antczak M, Zablocki M, Zok T, et al. RNAvista: a webserver to assess RNA secondary structures with non-canonical base pairs. Bioinformatics 2019;35:152–5. 29985979 10.1093/bioinformatics/bty609 PMC6298044

[231] Doshi KJ, Cannone JJ, Cobaugh CW, Gutell RR. Evaluation of the suitability of free-energy minimization using nearest-neighbor energy parameters for RNA secondary structure prediction. BMC Bioinformatics 2004;5:105. 15296519 10.1186/1471-2105-5-105 PMC514602

[232] DiChiacchio L, Mathews DH. Predicting RNA-RNA interactions using RNAstructure. Methods Mol Biol 2016;1490:51–62. 27665592 10.1007/978-1-4939-6433-8_4

[233] Zhao Q, Zhao Z, Fan X, et al. Review of machine learning methods for RNA secondary structure prediction. PLoS Comput Biol 2021;17:e21009291. 10.1371/journal.pcbi.1009291 PMC8389396 34437528

[234] Sato K, Hamada M. Recent trends in RNA informatics: a review of machine learning and deep learning for RNA secondary structure prediction and RNA drug discovery. Brief Bioinform 2023;24:bbad186. 37232359 10.1093/bib/bbad186 PMC10359090

[235] Justyna M, Antczak M, Szachniuk M. Machine learning for RNA 2D structure prediction benchmarked on experimental data. Brief Bioinform 2023;24:bbad153. 37096592 10.1093/bib/bbad153 PMC10199776

[236] Le Quy T, Roy A, Iosifidis V, et al. A survey on datasets for fairness-aware machine learning. WIREs Data Mining Knowledge Discov 2022;12:e1452.

[237] Townshend RJL, Eismann S, Watkins AM, et al. Geometric deep learning of RNA structure. Science 2021;373:1047–51. 34446608 10.1126/science.abe5650 PMC9829186

[238] Zhang Y, Liu T, Chen L, et al. RIscoper: a tool for RNA–RNA interaction extraction from the literature. Bioinformatics 2019;35:3199–202. 30668649 10.1093/bioinformatics/btz044

[239] Chen Q, Leaman R, Allot A, et al. Artificial intelligence in action: addressing the COVID-19 pandemic with natural language processing. Ann Rev Biomed Data Sci 2021;4:313–39. 34465169 10.1146/annurev-biodatasci-021821-061045

[240] JWJ A, Tataru P, Staines J, et al. Evolving stochastic context-free grammars for RNA secondary structure prediction. BMC Bioinformatics 2012;13:78. 22559985 10.1186/1471-2105-13-78 PMC3464655

[241] Andronescu M, Condon A, Hoos HH, et al. Efficient parameter estimation for RNA secondary structure prediction. Bioinformatics 2007;23:i19–28. 17646296 10.1093/bioinformatics/btm223

[242] Do CB, Woods DA, Batzoglou S. CONTRAfold: RNA secondary structure prediction without physics-based models. Bioinformatics 2006;22:e90–8. 16873527 10.1093/bioinformatics/btl246

[243] Zakov S, Goldberg Y, Elhadad M, Ziv-ukelson M. Rich parameterization improves RNA structure prediction. J Comput Biol 2011;18:1525–42. 22035327 10.1089/cmb.2011.0184

[244] Rivas E, Lang R, Eddy SR. A range of complex probabilistic models for RNA secondary structure prediction that includes the nearest-neighbor model and more. RNA (New York, NY) 2012;18:193–212. 10.1261/rna.030049.111 PMC3264907 22194308

[245] Yu H, Qi Y, Ding Y. Deep learning in RNA structure studies. Front Mol Biosci 2022;9:869601. 35677883 10.3389/fmolb.2022.869601 PMC9168262

[246] Singh J, Hanson J, Paliwal K, Zhou Y. RNA secondary structure prediction using an ensemble of two-dimensional deep neural networks and transfer learning. Nat Commun 2019;10:5407. 31776342 10.1038/s41467-019-13395-9 PMC6881452

[247] Wang L, Liu Y, Zhong X, et al. DMfold: a novel method to predict RNA secondary structure with pseudoknots based on deep learning and improved base pair maximization principle. Front Genet 2019;10:243. 10.3389/fgene.2019.00143 PMC6409321 30886627

[248] Górska A, Jasiński M, Trylska J. MINT: software to identify motifs and short-range interactions in trajectories of nucleic acids. Nucleic Acids Res 2015;43:e114–4. 26024667 10.1093/nar/gkv559 PMC4787793

[249] Puton T, Kozlowski LP, Rother KM, Bujnicki JM. CompaRNA: a server for continuous benchmarking of automated methods for RNA secondary structure prediction. Nucleic Acids Res 2014;42:5403–6. 24682823 10.1093/nar/gku208 PMC4005657

[250] Puton T, Kozlowski LP, Rother KM, Bujnicki JM. CompaRNA: a server for continuous benchmarking of automated methods for RNA secondary structure prediction. Nucleic Acids Res 2013;41:4307–23. 23435231 10.1093/nar/gkt101 PMC3627593

[251] Umu SU, Poole AM, Dobson RC, et al. Avoidance of stochastic RNA interactions can be harnessed to control protein expression levels in bacteria and archaea. Elife 5:e13479. 10.7554/eLife.13479 PMC5028192 27642845

[252] Will S, Joshi T, Hofacker IL, et al. LocARNA-P: accurate boundary prediction and improved detection of structural RNAs. RNA 2012;18:900–14. 22450757 10.1261/rna.029041.111 PMC3334699

[253] Siebert S, Backofen R. MARNA: multiple alignment and consensus structure prediction of RNAs based on sequence structure comparisons. Bioinformatics 2005;21:3352–9. 15972285 10.1093/bioinformatics/bti550

[254] Higgs PG . RNA secondary structure: physical and computational aspects. Q Rev Biophys 2000;33:199–253. 11191843 10.1017/s0033583500003620

[255] Klein RJ, Eddy SR. RSEARCH: finding homologs of single structured RNA sequences. BMC Bioinformatics 2003;4:44. 14499004 10.1186/1471-2105-4-44 PMC239859

[256] Mamuye A, Merelli E, Tesei L. A graph grammar for modelling RNA folding. Electron Proc Theor Comput Sci 2016;231:31–41.

[257] Pervouchine DD . Towards long-range RNA structure prediction in eukaryotic genes. Genes 2018;9:302. 29914113 10.3390/genes9060302 PMC6027157

[258] Fukunaga T, Iwakiri J, Ono Y, Hamada M. LncRRIsearch: a web server for lncRNA-RNA interaction prediction integrated with tissue-specific expression and subcellular localization data. Front Genet 2019;10:462. 10.3389/fgene.2019.00462 PMC6546843 31191601

[259] Reuter JS, Mathews DH. RNAstructure: software for RNA secondary structure prediction and analysis. BMC Bioinformatics 2010;11:129. 20230624 10.1186/1471-2105-11-129 PMC2984261

[260] Reynolds A, Leake D, Boese Q, et al. Rational siRNA design for RNA interference. Nat Biotechnol 2004;22:326–30. 14758366 10.1038/nbt936

[261] Rennie W, Kanoria S, Liu C, et al. Sfold Tools for MicroRNA Target Prediction. Methods Mol Biol 2019;1970:31–42. 30963486 10.1007/978-1-4939-9207-2_3

[262] DiChiacchio L, Sloma MF, Mathews DH. AccessFold: predicting RNA–RNA interactions with consideration for competing self-structure. Bioinformatics 2016;32:1033–9. 26589271 10.1093/bioinformatics/btv682 PMC4907385

[263] Wuchty S, Fontana W, Hofacker IL, Schuster P. Complete suboptimal folding of RNA and the stability of secondary structures. Biopolymers 1999;49:145–65. 10070264 10.1002/(SICI)1097-0282(199902)49:2<145::AID-BIP4>3.0.CO;2-G

[264] Tan Z, Fu Y, Sharma G, Mathews DH. TurboFold II: RNA structural alignment and secondary structure prediction informed by multiple homologs. Nucleic Acids Res 2017;45:11570–81. 29036420 10.1093/nar/gkx815 PMC5714223

[265] Felsenstein J . Evolutionary trees from DNA sequences: a maximum likelihood approach. J Mol Evol 1981;17:368–76. 7288891 10.1007/BF01734359

[266] Durbin R, Eddy SR, Krogh A, et al. Chapter 9: Transformational grammars. In: Biological Sequence Analysis: Probabilistic Models of Proteins and Nucleic Acidssource. New York, USA: Cambridge University Press, 1998, pp. 233–59.

[267] Fu Y, Sharma G, Mathews DH. Dynalign II: common secondary structure prediction for RNA homologs with domain insertions. Nucleic Acids Res 2014;42:13939–48. 25416799 10.1093/nar/gku1172 PMC4267632

[268] Xu Z, Mathews DH. Multilign: an algorithm to predict secondary structures conserved in multiple RNA sequences. Bioinformatics 2011;27:626–32. 21193521 10.1093/bioinformatics/btq726 PMC3042186

[269] Sorescu DA, Möhl M, Mann M, et al. CARNA—alignment of RNA structure ensembles. Nucleic Acids Res 2012;40:W49–53. 22689637 10.1093/nar/gks491 PMC3394245

[270] Horesh Y, Doniger T, Michaeli S, Unger R. RNAspa: a shortest path approach for comparative prediction of the secondary structure of ncRNA molecules. BMC Bioinformatics 2007;8:366. 17908318 10.1186/1471-2105-8-366 PMC2147038

[271] Ji Y, Xu X, Stormo GD. A graph theoretical approach for predicting common RNA secondary structure motifs including pseudoknots in unaligned sequences. Bioinformatics 2004;20:1591–602. 14962926 10.1093/bioinformatics/bth131

[272] Winkler J, Urgese G, Ficarra E, Reinert K. LaRA 2: parallel and vectorized program for sequence–structure alignment of RNA sequences. BMC Bioinformatics 2022;23:18. 34991448 10.1186/s12859-021-04532-7 PMC8734264

[273] Hochsmann M, Toller T, Giegerich R, et al. Local similarity in RNA secondary structures. In: Computational Systems Bioinformatics. Proceedings of the 2003 IEEE Bioinformatics Conference. Stanford, CA, 2003, pp. 159–68. The Institute of Electrical and Electronics Engineers, New York, USA. 16452790

[274] Raden M, Ali SM, Alkhnbashi OS, et al. Freiburg RNA tools: a central online resource for RNA-focused research and teaching. Nucleic Acids Res 2018;46:W25–9. 29788132 10.1093/nar/gky329 PMC6030932

[275] Reeder J, Steffen P, Giegerich R. pknotsRG: RNA pseudoknot folding including near-optimal structures and sliding windows. Nucleic Acids Res 2007;35:W320–4. 17478505 10.1093/nar/gkm258 PMC1933184

[276] Macke TJ, Ecker DJ, Gutell RR, et al. RNAMotif, an RNA secondary structure definition and search algorithm. Nucleic Acids Res 2001;29:4724–35. 11713323 10.1093/nar/29.22.4724 PMC92549

[277] Legendre A, Angel E, Tahi F. RCPred: RNA complex prediction as a constrained maximum weight clique problem. BMC Bioinformatics 2019;20:128. 30925864 10.1186/s12859-019-2648-1 PMC6439972

[278] Bindewald E, Afonin KA, Viard M, et al. Multistrand structure prediction of nucleic acid assemblies and design of RNA switches. Nano Lett 2016;16:1726–35. 26926528 10.1021/acs.nanolett.5b04651 PMC6319913

[279] Xu X, Zhao P, Chen S-J. Vfold: a web server for RNA structure and folding thermodynamics prediction. PloS One 2014;9:e107504. 25215508 10.1371/journal.pone.0107504 PMC4162592

[280] Bellaousov S, Mathews DH. ProbKnot: fast prediction of RNA secondary structure including pseudoknots. RNA 2010;16:1870–80. 20699301 10.1261/rna.2125310 PMC2941096

[281] Krogh A, Brown M, Mian IS, et al. Hidden Markov models in computational biology. Applications to protein modeling. J Mol Biol 1994;235:1501–31. 8107089 10.1006/jmbi.1994.1104

[282] Sükösd Z, Andersen ES, Lyngsø R. SCFGs in RNA secondary structure prediction RNA secondary structure prediction: a hands-on approach. Methods Mol Biol 2014;1097:143–62. 24639159 10.1007/978-1-62703-709-9_8

[283] Darty K, Denise A, Ponty Y. VARNA: interactive drawing and editing of the RNA secondary structure. Bioinformatics 2009;25:1974–5. 19398448 10.1093/bioinformatics/btp250 PMC2712331

[284] Willmott D, Murrugarra D, Ye Q. Improving RNA secondary structure prediction via state inference with deep recurrent neural networks. Comput Mathemat Biophys 2020;8:36–50.

[285] Deschenes A, Wiese KC, Poonian J. Comparison of dynamic programming and evolutionary algorithms for RNA secondary structure prediction. In: 2004 IEEE International Geoscience and Remote Sensing (IEEE Cat. No.04CH37612). Anchorage, AK, USA, 2004, 214–22. The Institute of Electrical and Electronics Engineers, New York, USA.

